# Effectiveness and economic assessment of routine larviciding for prevention of chikungunya and dengue in temperate urban settings in Europe

**DOI:** 10.1371/journal.pntd.0005918

**Published:** 2017-09-11

**Authors:** Giorgio Guzzetta, Filippo Trentini, Piero Poletti, Frederic Alexandre Baldacchino, Fabrizio Montarsi, Gioia Capelli, Annapaola Rizzoli, Roberto Rosà, Stefano Merler, Alessia Melegaro

**Affiliations:** 1 Fondazione Bruno Kessler, Trento, Italy; 2 Dondena Centre for Research on Social Dynamics and Public Policy, Bocconi University, Milan, Italy; 3 Department of Biodiversity and Molecular Ecology, Fondazione Edmund Mach, San Michele all'Adige (Trento), Italy; 4 Laboratory of Parasitology, Istituto Zooprofilattico Sperimentale delle Venezie, Padova, Italy; 5 Department of Policy Analysis and Public Management, Bocconi University, Milan, Italy; Faculty of Science, Mahidol University, THAILAND

## Abstract

In the last decades, several European countries where arboviral infections are not endemic have faced outbreaks of diseases such as chikungunya and dengue, initially introduced by infectious travellers from tropical endemic areas and then spread locally via mosquito bites. To keep in check the epidemiological risk, interventions targeted to control vector abundance can be implemented by local authorities. We assessed the epidemiological effectiveness and economic costs and benefits of routine larviciding in European towns with temperate climate, using a mathematical model of *Aedes albopictus* populations and viral transmission, calibrated on entomological surveillance data collected from ten municipalities in Northern Italy during 2014 and 2015.We found that routine larviciding of public catch basins can limit both the risk of autochthonous transmission and the size of potential epidemics. Ideal larvicide interventions should be timed in such a way to cover the month of July. Optimally timed larviciding can reduce locally transmitted cases of chikungunya by 20% - 33% for a single application (dengue: 18–22%) and up to 43% - 65% if treatment is repeated four times throughout the season (dengue: 31–51%). In larger municipalities (>35,000 inhabitants), the cost of comprehensive larviciding over the whole urban area overcomes potential health benefits related to preventing cases of disease, suggesting the adoption of more localized interventions. Small/medium sized towns with high mosquito abundance will likely have a positive cost-benefit balance. Involvement of private citizens in routine larviciding activities further reduces transmission risks but with disproportionate costs of intervention. International travels and the incidence of mosquito-borne diseases are increasing worldwide, exposing a growing number of European citizens to higher risks of potential outbreaks. Results from this study may support the planning and timing of interventions aimed to reduce the probability of autochthonous transmission as well as the nuisance for local populations living in temperate areas of Europe.

## Author summary

Larvicides are a key tool to prevent the growth of mosquito populations and decrease both the risks of outbreaks of mosquito-borne diseases and the nuisance deriving from bites. In order to assist municipalities from temperate areas in Europe in effectively planning vector control programs, we modelled the effect of larviciding in public areas on populations of *Aedes albopictus* using mosquito collection data from 10 municipalities in Northern Italy, over two years with very different temperature conditions. We then evaluated the resulting probabilities of potential outbreaks of chikungunya and dengue and their expected final sizes, and we compared the intervention costs to the economic and health benefits due to the avoidance of clinical cases. By assessing several different intervention strategies, we found that the optimal timing should be centred on the month of July, corresponding to the period of maximal growth of the mosquito population. Municipality-wide interventions are likely to be cost-effective in small-to-medium towns (below 35,000 inhabitants) even where mosquito infestation is moderate, whereas for larger cities a neighbourhood-based intervention should be considered. The involvement of citizens to apply larvicides within private premises resulted effective but generally too costly.

## Introduction

During the last decade, Europe has faced outbreaks of mosquito-borne diseases (MBD) such as dengue and chikungunya, following the continuous importation of human cases in areas with established competent vectors such as the invasive mosquito *Aedes* (*Stegomyia*) *albopictus* (Skuse) [1]. Vector control interventions can be implemented by local authorities to keep in check mosquito abundance and consequently reduce the epidemiological risk. Adulticide spraying rapidly reduces the number of mosquitoes, but its effect is short-lived [2]. For this reason, it is particularly indicated in situations where the transmission risk needs to be reduced drastically and quickly, such as when an individual is diagnosed with an MBD, to prevent or curtail an outbreak [3]. Since the effectiveness of reactive measures decreases with the delay between outbreak initiation and implementation of control [4], a better approach may consist in preventive interventions. Treatment of potential breeding sites with larvicide products has a delayed impact in reducing adult populations [3], but experimental studies show that their effect lasts for several weeks [5], making them better suited for preventive routine control. The main limit to larviciding as a control option is the proportion of breeding sites that are actually accessible to interventions by public health authorities. To overcome this limit, education campaigns may be carried forward to encourage citizens to remove and treat potential breeding sites from their private premises during the mosquito season [6, 7]. Mathematical modelling of MBD associated with cost-effectiveness analyses can help optimizing routine vector control interventions [8] with respect to constraints in human and financial resources [9].

With the aim of assisting European municipalities in planning and timing preventive vector control, we assessed the potential epidemiological impact on chikungunya and dengue, and the ensuing economic benefits for the health system, produced by routine larviciding against *Ae*. *albopictus* within urban sites in temperate climates.

## Methods

### Mosquito data

Mosquito monitoring via adult trapping was carried out in ten municipalities from the Northern Italian provinces of Belluno and Trento, characterized by a temperate climate [10]. Mosquitoes were collected using Biogents (BG) Sentinel traps (Biogents AG, Regensburg, Germany) baited with lures and CO_2_ from dry ice. After each trapping session, mosquitoes were killed by freezing at -20°C, identified using taxonomic keys [11, 12] and confirmed by PCR if found in a location for the first time [12].

### Transmission dynamics model

We simulated the transmission dynamics associated with chikungunya and dengue using a standard SEI-SEIR approach [13] in which mosquitoes develop lifelong infection after an (extrinsic) incubation period since the bite to an infectious human (SEI sub-model), whereas humans develop temporary infection, followed by the development of immunity, after an (intrinsic) incubation period since the bite from an infectious mosquito (SEIR sub-model). We considered temperature-dependent extrinsic incubation periods and per-bite transmission probabilities for dengue [14], whereas only temperature-independent estimates were available for Chikungunya [15, 16]. The transmission model was initialized with a single infectious human, representing an imported case at a date sampled uniformly between January 1^st^ and December 31^st^. The population size of female *Ae*. *albopictus* mosquitoes over time in the transmission model was estimated by fitting a population model to capture data collected in the absence of larvicidal treatments, following the same approach already adopted in [13, 17]. The model considers four developmental stages of mosquitoes (eggs, larvae, pupae and adults) and reproduces their life cycle by means of temperature-dependent parameters regulating the stage-specific rates of mortality and development. Free model parameters (i.e. the site- and year- specific habitat suitability and the capture rate of BG traps) were estimated via a Monte Carlo Markov Chain approach based on a Poisson likelihood [13, 17].

We then included the effect of routine larviciding in the population model. Experimental studies of several available commercial larvicide products show that 99% of existing larvae and hatching eggs are killed within a given breeding site, with constant efficacy of about 30 days, independently of the specific product used [5, 18, 19]. We considered a standard approach targeting breeding sites in publicly accessible spaces (e.g., catch basins placed in public parks and along the road system), and an additional strategy where public interventions were integrated by the involvement of citizens in treating and removing breeding sites within private premises. The latter was parametrized on results from a pilot project conducted in two municipalities within the same area of this study [7], in which larvicide products were delivered door-to-door and free of charge to house dwellers, who were sensitized and educated to mosquito control interventions. A key determinant of effectiveness is the fraction of existing breeding sites in a given area that are actually treated (coverage). We adopted a coverage range between 30% and 50% for larviciding of public catch basins only, and between 60% and 75% for interventions that additionally involve citizens. These ranges were computed from available data on the density and proportion of reachable breeding sites in public and private premises [7]. Other strategies aimed at extending the coverage (e.g. removal of other breeding sites such as water buckets, plant saucers, tarpaulins, etc.) were not considered.

Since the effect of larvicides is transitory, treatment of catch basins may be repeated multiple times within a given season. We considered several different starting dates and from 1 to 4 applications of larvicide treatments within a given mosquito season (hereafter referred to as “effort level”), implemented with monthly frequency.

### Economic assessment

To evaluate the economic acceptability of the two considered strategies, a cost-utility analysis for the prevention of dengue and chikungunya was conducted, taking the number of infections as input from the transmission model. Disability Adjusted Life Years (DALYs) averted and net costs were derived comparing an intervention scenario to the case in which no control programs were put in place (baseline). The baseline was set to reflect a municipality where only the monitoring of mosquito presence via ovitraps was performed [7].

The analysis was conducted from a public healthcare system perspective through the maximization of the net health benefit (NHB) [20]. This measure is defined as the difference between the DALY averted and the incremental cost due to the intervention, the latter divided by the willingness to pay (WTP) by public authorities for each DALY averted. Following WHO recommendations [21], we assumed such value approximately equal to the Gross Domestic Product (GDP), which is about 35,000 euro per capita in our study area [22].

Probabilities of each infected case of being symptomatic, notified, severe, hospitalized and of dying, and the length of stay in hospital, were derived from published studies [23, 24] and from analyzing data from the Italian Hospital Discharge System (Schede di Dimissioni Ospedaliere), accounting for all hospital admissions for chikungunya and dengue recorded in Italy. The cost of illness was estimated according to expert opinion. The costs of intervention were estimated from actual costs during control activities against *Ae*. *albopictus* recently performed in two municipalities from the study area [7].

For all the considered scenarios, the NHB was computed on a set of 100,000 stochastic realizations accounting for the uncertainty in both the transmission and the economic model’s parameters. Full details on this analysis are provided in S1 Text.

To assess the feasibility and sustainability of public interventions, we used responses from a questionnaire administered in 2013 to municipalities of the province of Trento, aimed at collecting information on the actual public expenditure on vector control activities.

## Results

**Fig 1:**
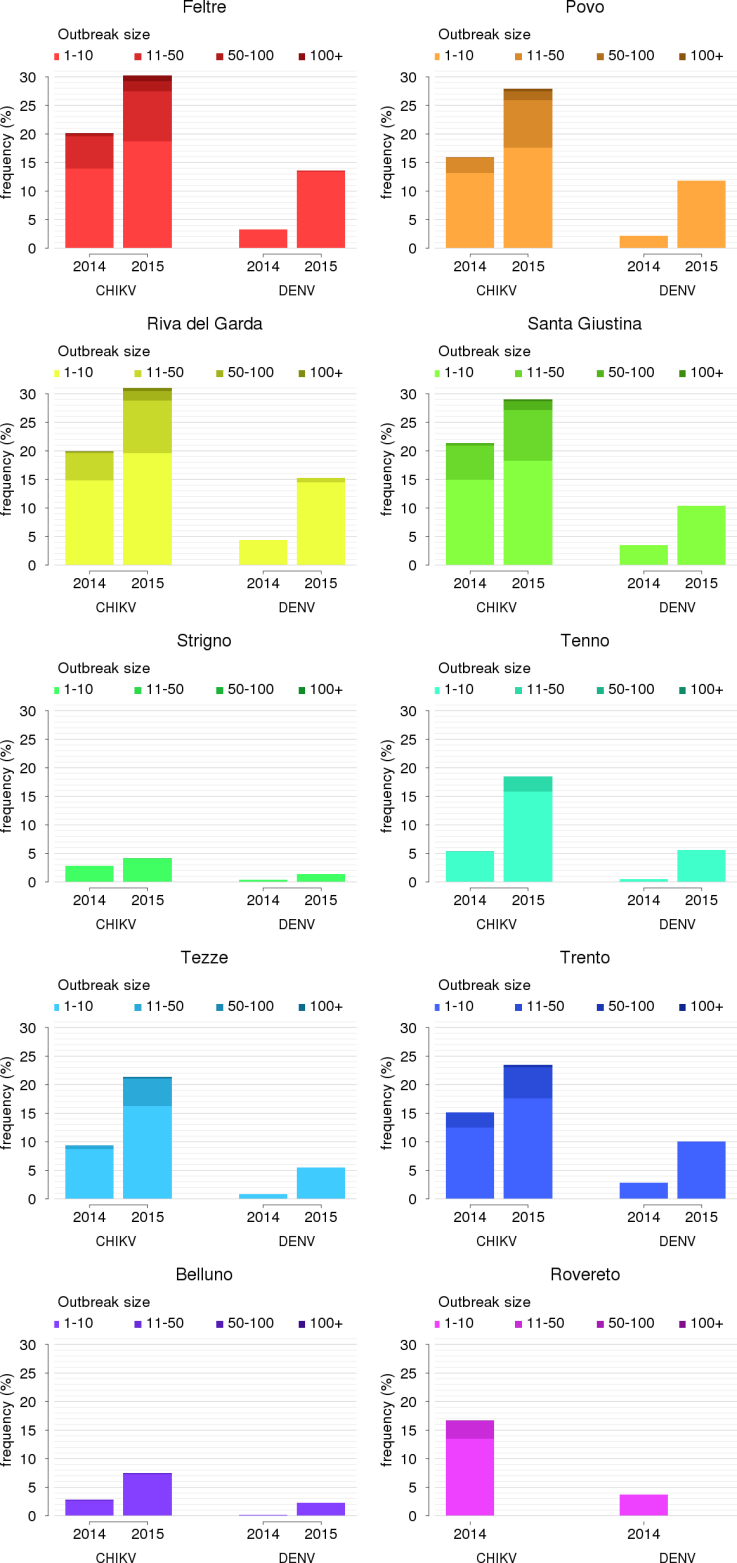
Epidemiological predictions in the absence of control interventions. Probability of local transmission of chikungunya (CHIKV) and dengue (DENV), disaggregated by outbreak size, in the 10 study locations during the mosquito seasons.

The estimated density of adult female mosquitoes (averaged between April 10^th^ and September 30^th^) was between 4 and 88 per hectare in 2014 and between 9 and 198 in 2015, depending on the municipality (see Table 1). The higher abundance in 2015 is mostly due to the much higher temperatures recorded during summer. The initial reproduction numbers and the threshold for autochthonous transmission of chikungunya and dengue over time were estimated in a previous study [17]. Here, for each site and year, we computed the probability of autochthonous transmission of chikungunya and dengue originated by an imported infection in the absence of interventions. Higher vector densities during 2015 resulted in an increased risk of local transmission for both infections, compared to the previous year. The probability of observing at least one secondary case was estimated to be up to 30% for chikungunya and 15% for dengue in highly infested towns in 2015. Corresponding maximum probabilities in 2014 were around 20% for chikungunya and 5% for dengue. This means that 7 importations of chikungunya and 15 importations of dengue in towns most at risk would have a >90% probability of causing at least one secondary case in 2015. Sporadic transmission (less than 10 secondary cases) is by far the most likely scenario, especially for dengue (Fig 1). However, we found a low, but non-negligible, probability (up to 2.7%) that an uncontrolled chikungunya outbreak would produce more than 50 cases in several sites during 2015.

**Table 1:** Estimated mosquito densities (average between April 10^th^–September 30^th^) and other relevant characteristics of the considered municipalities.

Municipality	Mosquito density (adult mosquitoes / ha)		Population (inhabitants)	Urban surface (ha)	Population density (inhabitants / ha)
	2014	2015			
**Strigno** *	3.8	8.9	3354	85	39.5
**Belluno**	9.8	27.1	35703	539	66.2
**Tenno**	14.1	88.9	1823	23	79.3
Tezze**	19.9	72.1	1742	36	48.4
**Povo**	42.4	138.5	5571	88	63.3
**Santa Giustina**	46.4	95.6	6800	159	42.8
**Trento**	52.3	128.4	117304	1570	74.7
**Riva del Garda** ***	72.7	197.5	32259	418	77.2
**Feltre**	74.2	161.1	20560	329	62.5
**Rovereto**	87.6	-	39099	444	88.1

*: includes the neighbouring municipality of Telve

**: includes the neighbouring municipality of Villa Agnedo

***: includes the neighbouring municipality of Arco

Routine preventive larvicide treatments can reduce significantly mosquito populations and consequently the probability and size of outbreaks triggered by sporadic importation of infected cases. To evaluate the overall effectiveness, we considered the expected number of total secondary infections per imported case. Under the baseline scenario of no control interventions, this index ranged from 0.1 to 5.2, depending on the site and year; corresponding numbers for dengue were everywhere below 0.5. Because of the smaller epidemiological risk of dengue, we discuss only the cost-effectiveness analysis on chikungunya, leaving corresponding results for dengue to the S1 Text.

**Fig 2:**
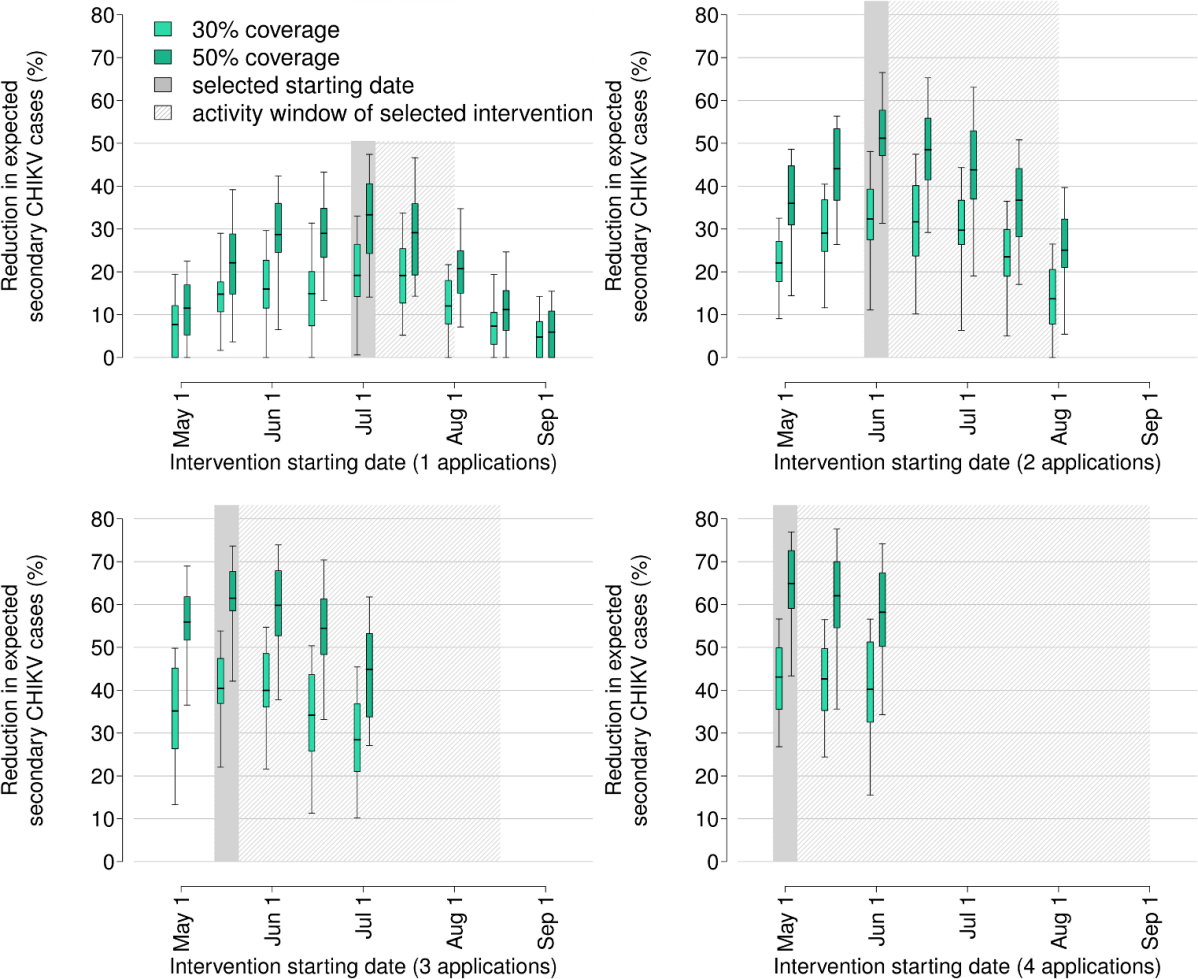
Effectiveness of larviciding in reducing the expected number of secondary CHIKV infections. Distribution across all sites and both years, disaggregated by coverage value (lighter colour: 30%; darker colour: 50%), intervention timing (starting date every 15 days between May 1^st^ and September 1^st^) and effort level (i.e., number of larvicide applications with monthly frequency (from 1 to 4). Grey bars: optimal starting date; shaded area: duration of intervention.

**Fig 3:**
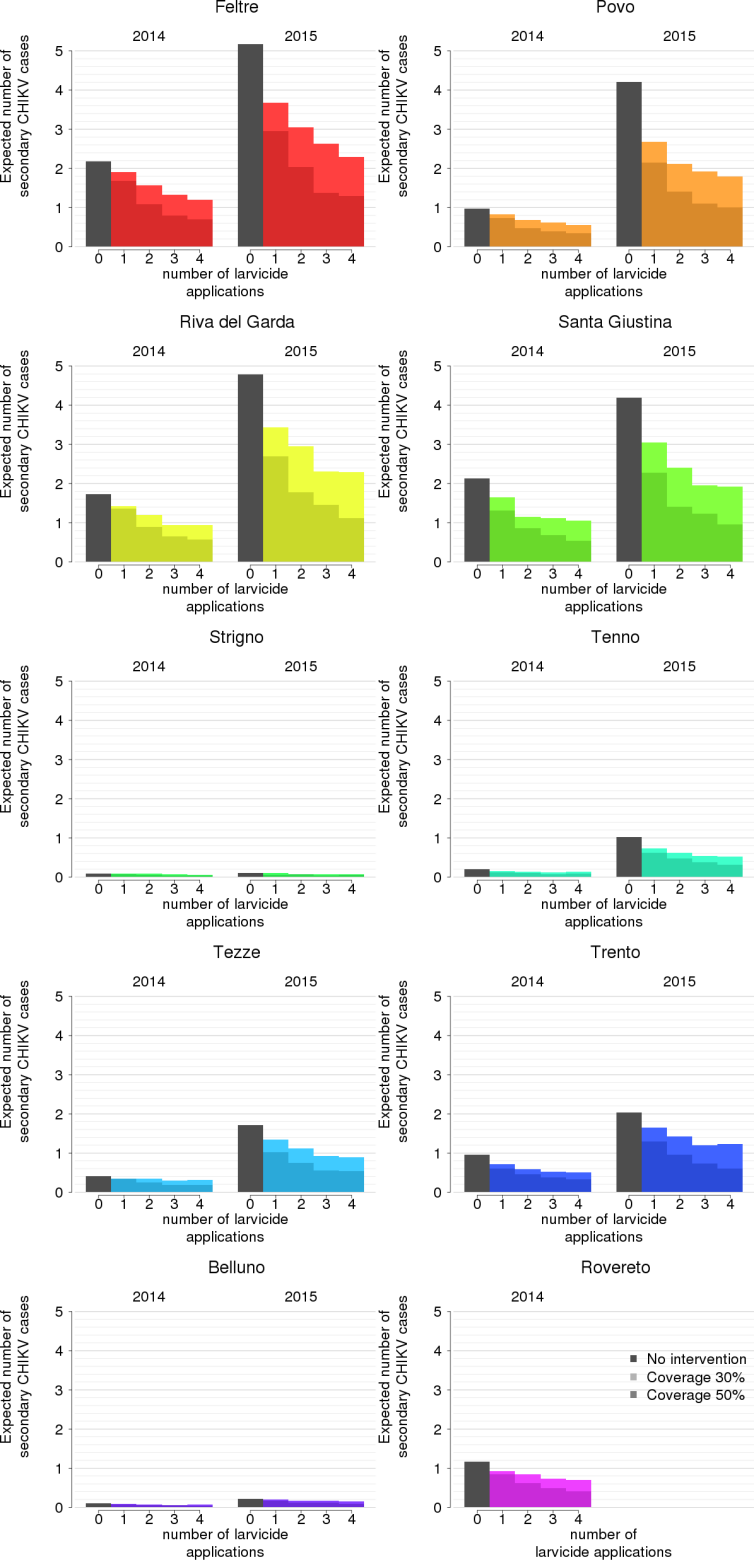
Estimated effectiveness of optimally timed interventions for different coverages (30% and 50%) and effort level (0–4), disaggregated by site and year.

For each site and year, and for each timing, effort level and assumed coverage, we evaluated the relative reduction in the expected number of secondary infections per imported case as a measure of effectiveness. Fig 2 and Table 2 show that all interventions with optimal effectiveness covered the month of July, which corresponds to the estimated period of steepest growth of the adult *Ae*. *albopictus* population in both years. We selected for further analyses only interventions with optimal timing for each effort level (Table 2; the reduction in mosquito abundance corresponding to the optimally timed interventions is reported in the S1 Text). We found that an increase in the effort level does not proportionally reduce the expected number of cases (Fig 3). In particular, an expansion in the coverage of breeding sites from 30% to 50% would be more effective than doubling the effort level while keeping the coverage at 30%. In general, interventions are most beneficial when the baseline risk is highest.

**Table 2:** Timing of optimal intervention and estimated reduction in the number of chikungunya cases at 30% and 50% coverage (mean and range across all sites) by effort level (one to four larvicide applications over a season).

Effort level	Optimal starting date	End of efficacy, optimal timing	Reduction in number of CHIKV cases, % (range)	
			30% coverage	50% coverage
**1 intervention**	July 1^st^	August 1^st^	18.6 (0–36.4) %	33.3 (13.9–48.9) %
**2 interventions**	June 1^st^	August 1^st^	32.3 (6.9–49.8) %	51.2 (27.7–66.6) %
**3 interventions**	May 15^th^	August 15^th^	40.5 (20.8–54.4) %	61.5 (39.0–74.0) %
**4 interventions**	May 1^st^	September 1^st^	43.1 (23.7–57.3) %	64.9 (39.2–77.2) %

**Fig 4:**
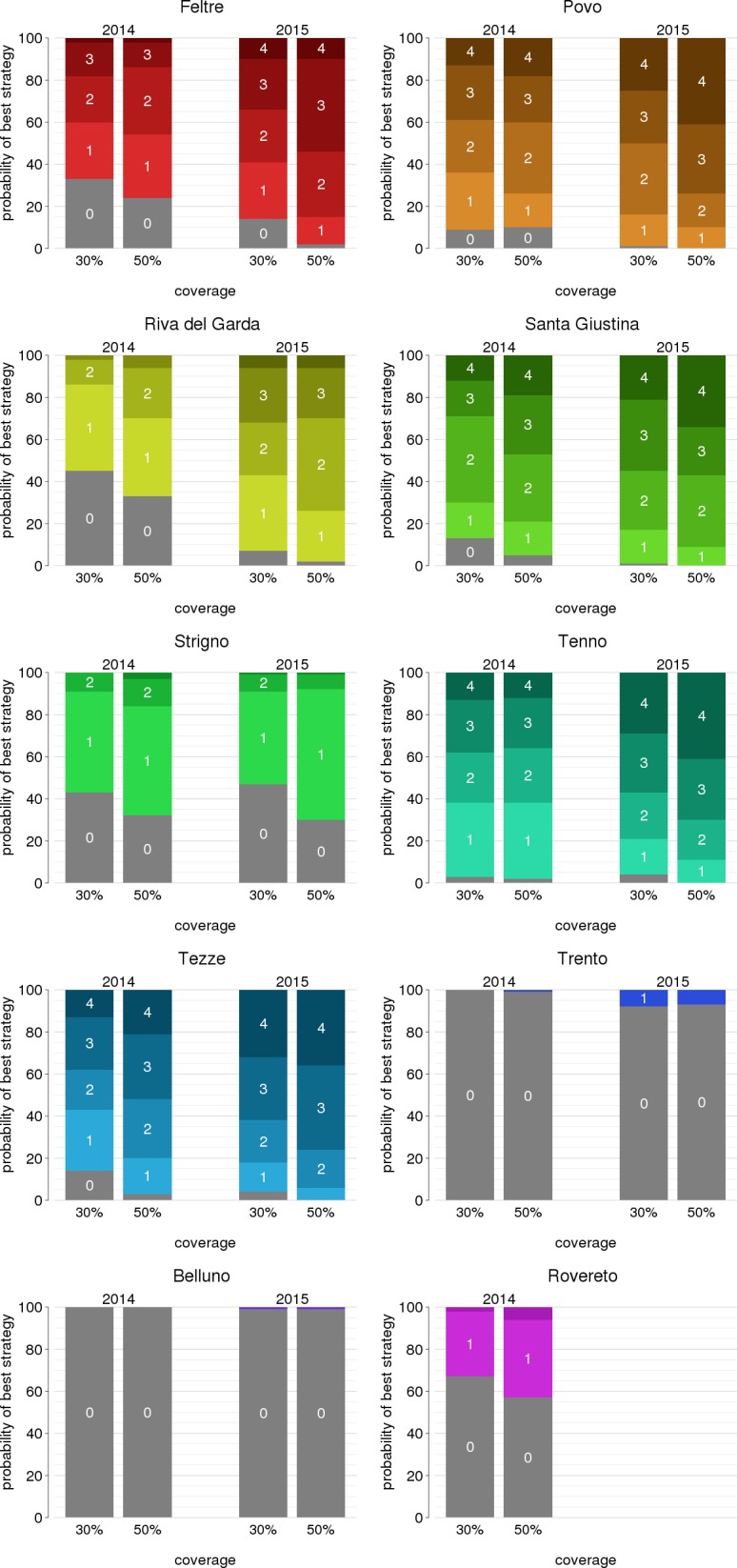
Probability of highest net health benefit according to the number of larvicide treatments, disaggregated by year, coverage and study site.

**Fig 5:**
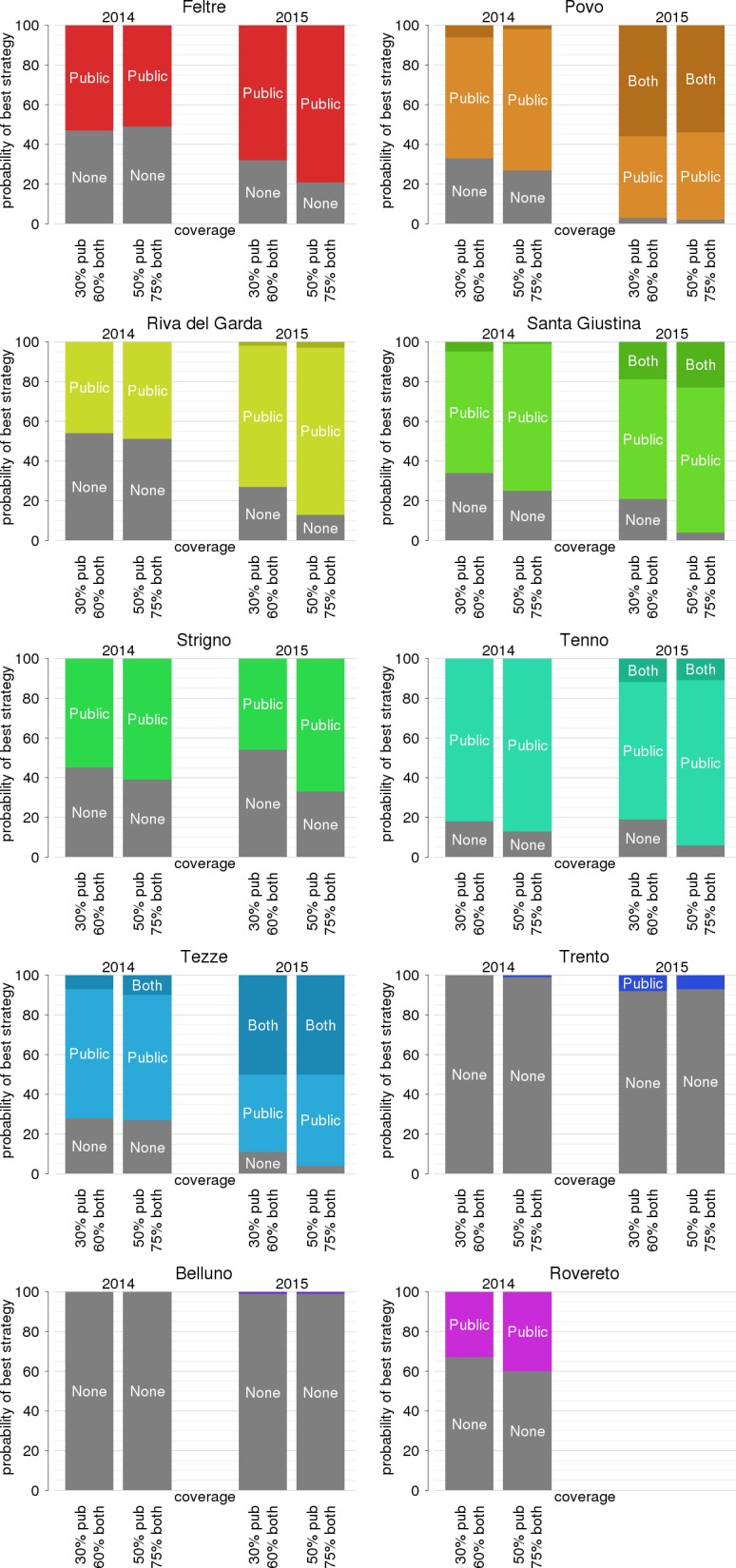
Probability of highest net health benefit for a single larvicide application according to the type of intervention (none vs. public vs. public and private), by year, coverage (30 and 50% for public intervention; 60 and 75% for both public and private intervention) and study site.

Towards an optimal allocation of resources, the benefits of reducing the potential number of transmitted cases needs to be compared with the intervention costs. Taking into account all possible clinical outcomes, including the probability of severe illness and of hospitalization, the estimated average cost per infection is 424.9 euros (95% CI 342–533) for chikungunya and 275.88 euros (95% CI 151–422) for each dengue infection. The corresponding average DALY loss per case is higher for chikungunya (0.45, 95% CI 0.10–1.12) than for dengue (0.29, 95% CI 0.15–0.44). In Fig 4, we show the relative probability that each effort level (including the no-intervention scenario) will maximize the NHB for each site, year, and coverage. Three main outcomes can be identified. The first is represented by larger cities (Trento, Belluno and Rovereto, all above 35,000 inhabitants) where non-intervention has the highest likelihood of being optimal. In these sites, the poor economic effectiveness of larviciding depends on the relatively low number of expected secondary cases even in the absence of treatment (Fig 3), combined with the high intervention costs due to the extent of the area to be covered. The second group consists of smaller towns where intervention is always beneficial (Povo, Santa Giustina, Tenno and Tezze, all below 10,000 inhabitants) and where higher effort levels have the highest probabilities of being optimal. Strigno (about 3,400 inhabitants) represents an exception to this rule, where the low intervention costs are counterbalanced by a very small transmission risk in the absence of interventions. Nonetheless, even in Strigno a low-effort intervention (single treatment) might be beneficial because of its low cost. The third situation occurs in towns of intermediate size (Feltre and Riva del Garda, between 20,000 and 35,000 inhabitants) where both the intervention costs and the transmission risks are high. In these cases, depending on the larviciding coverage, absence of intervention might be the optimal strategy in seasons of lower mosquito abundance (2014) while a low-to-moderate effort (1 to 3 treatments) might be the best choice in years of high infestation (2015). Overall, the probability that a more intensive intervention will be optimal increases with the coverage and with higher transmission risk (2015 vs. 2014). We also tested the cost-effectiveness of expanding the coverage by involving private citizens [7]. We found that this type of intervention might achieve significant additional reductions in the expected number of secondary cases and probability of local transmission (reported in the S1 Text). However, they are rarely optimal from the economic perspective because they require labour-intensive activities. Fig 5 reports results of the NHB analysis for a single larvicide treatment, but qualitative inferences are similar for more intensive efforts (see S1 Text). The only two instances where involvement of citizens was found to be economically beneficial were Povo and Tezze and only during the 2015 mosquito season, i.e. only where the urban size is small enough to keep intervention costs low and where the transmission risk at baseline is sufficiently high.

Two municipalities under study, Trento and Riva del Garda, had responded to a previously administered questionnaire on public expenditure on vector control, declaring an overall budget of 0.254 euro and 0.532 euros per inhabitant, respectively. In Trento, the most cost-effective activity predicted by our model was monitoring by ovitraps (Fig 4), which has an estimated average cost of 0.016 euro per inhabitant; in Riva del Garda, one or two larvicide applications per year would be likely optimal and would cost between 0.256 and 0.512 euros per inhabitant. Therefore, the most cost-effective strategies are sustainable with respect to the current allocated budget. We provide full details on questionnaires, municipality-specific answers and intervention costs in the S1 Text.

## Discussion

In this work, we evaluated the effect of routine larviciding against dengue and chikungunya, two viruses transmitted by bites of *Ae*. *albopictus* mosquitoes. We used data from two seasons of entomological surveillance in multiple sites from northern Italy to parametrize a mathematical model of mosquito population dynamics and control. The population model was coupled with a transmission dynamics model and a cost-effectiveness analysis to identify suitable routine vector control strategies for temperate climate municipalities in Europe. We found that, in the absence of interventions, the risk of autochthonous dengue transmission was low and limited to sporadic transmission in both years, because of the relatively low competence of European strains of *Ae*. *albopictus*. On the other hand, the risk of a chikungunya outbreak was estimated to be up to 30% in 2015, with a non-negligible probability of observing outbreaks larger than 50 cases in most sites.

We found that the most effective interventions in reducing the amount of expected locally transmitted cases were those for which the window of larvicide efficacy covered at least the month of July (Fig 2, Table 2). Larviciding reduced the probability of secondary cases only moderately, but it had an important impact in avoiding larger outbreaks. Our analysis included two seasons that were representative of a broad range of mosquito abundances, due to the remarkable temperature differences. The cost-effectiveness of larviciding depends on the actual mosquito abundance in a given year; however, general rules could be identified independently of the considered year: small villages (<10,000 inhabitants) with moderate-to-high mosquito abundances will maximally benefit of intense larviciding efforts made of season-round monthly treatment of public catch basins. For medium-sized towns (20–35,000 inhabitants) with high infestation rate, the benefits are partially offset by the higher cost of intervention; in these cases, a moderate larviciding effort (1 to 3 treatments within the season) is recommended. Larger cities in our study (>35,000 inhabitants) were characterized by a low or intermediate transmission risk, and the high costs of an intervention covering the entire urban area made it economically disadvantageous. In these situations, treating specific neighbourhoods with highest mosquito abundance (called ‘hot spot' approach [25]) may be cost-effective. In order to evaluate such a scenario, however, it would be necessary to model the complex effect of the urban layout on the spatial distribution of breeding sites and on the dynamics of mosquito populations [7], which is out of the scope of our study. Treatment of private breeding sites via the direct involvement of citizens by door-to-door visits was recommended only in small towns with high mosquito infestation. A survey on the allocated budget for mosquito control programs across different municipalities showed that expenses required for the most cost-effective interventions are sustainable for the considered area.

These results need to be contextualized with respect to our simplifying assumptions. First, all results are given conditionally on a uniform probability of importation of an infectious individual within a given epidemiological year. For comparison, in the considered provinces of Trento and Belluno, three imported cases of dengue and one imported case of chikungunya were recorded in 2014 (C. Rizzo, personal communication); however, the actual importation rate may vary significantly by year and time of the year, depending on spatio-temporal patterns of global epidemics and international travel. We did not consider reactive interventions that are implemented when a case of chikungunya or dengue infection is detected or after an outbreak has started (e.g., insecticide air spraying in the neighbourhood of the index case [26]). In addition, our results are relative to the prevention of arboviral transmission; however, there may be other purposes in vector control activities, such as the reduction of nuisance for citizens, which were not included in our analysis. For what concerns the economic assessment, we did not consider the impact of local transmission detection on the blood supply chain. Upon clinical confirmation of a locally transmitted arboviral infection, restrictions on the usage of blood bags collected in the region are applied to prevent transmission via transfusions, and screening tests on available blood supplies are implemented [26]. These additional interventions are quite expensive, and savings associated to the reduction of transmission risk granted by larvicides may dramatically offset the cost-benefit balance in favour of the intervention. However, these costs are difficult to estimate because of the lack of sufficient data.

We did not include other arboviroses transmitted by *Ae*. *albopictus* because of their lower epidemiological relevance to the considered area. For example, the risk of Zika virus transmission was found to be close to zero in the study region, even under conservative scenarios [17]. Nonetheless, we note that larvicides produce simultaneous benefits in preventing multiple diseases transmitted not only by *Ae*. *albopictus* but also by other affected mosquito species (e.g. West Nile virus associated to *Culex pipiens* L.). Furthermore, larviciding may assist in limiting the spread of other invasive mosquito species such as *Aedes* (*Hulecoeteomyia*) *japonicus* (Theobald) and *Aedes* (*Hulecoeteomyia*) *koreicus* (Edwards) [1, 27]. An interesting research question is how the balance of ecological interactions between mosquito species [28] may be offset by such interventions.

Other studies [2, 6, 7] have investigated the effectiveness of vector control in Europe using different approaches. The cost-effectiveness of larvicidal treatment against *Ae*. *albopictus* in temperate climates has been evaluated only in combination with other interventions during an ongoing outbreak [29, 30]; other studies were based on endemic (extra-European) settings where transmission is mainly mediated by *Aedes (Stegomyia) aegypti* (Linneus) [31, 32]. Overall, results from different studies and approaches, including our own, are consistent in highlighting the potential of larviciding towards reducing mosquito populations; however, this reduction will not result in a complete elimination of the risk of local chikungunya or dengue transmission. Additional strategies may integrate the control of risks from mosquito-borne diseases, including source reduction methods (e.g. identification and removal of breeding sites), mass trapping (e.g. via lethal ovitraps) and approaches leveraging ecological interactions (such as the use of *Wolbachia* bacteria or the release of genetically sterilized male mosquitoes). A comprehensive review of the potential for these strategies can be found in [9], but specific cost-effectiveness studies are needed to identify optimal strategies for vector control. European municipalities with temperate climate where *Ae*. *albopictus* is established may take advantage of results from this study when planning and timing routine larviciding interventions aimed to prevent or reduce epidemiological risks. Temperate European areas share with our study collection area similar temperature suitability for the transmission of arboviruses [33] and similar abundances of *Ae*. *albopictus* [34], so that results on the epidemiological effectiveness of larviciding should not differ significantly. More caution should be paid when extrapolating cost-effectiveness conclusions to different countries, given potential differences in health and intervention costs and in the choice of the WTP. Finally, we suggest that the proposed methodological approach may also be extended to European areas with different climates, conditional on the availability of local data on mosquito abundances estimated via entomological surveillance activities.

## Supporting information

S1 Text Technical details on the implementation of the transmission dynamics model, the cost-effectiveness analysis and additional model results. (PDF)

S1 Dataset Capture data and daily temperatures for the 10 municipalities. (CSV)
