# Supplementary material for: Effectiveness and economic assessment of routine larviciding for prevention of chikungunya and dengue in temperate urban settings in Europe
Source: PLoS Negl Trop Dis. 2017 Sep 11;11(9):e0005918. doi: 10.1371/journal.pntd.0005918 (PMC5608415; doi:10.1371/journal.pntd.0005918)
Supplement: S1 Text — (PDF) [file pntd.0005918.s001.pdf]

Supplementary Materials

Effectiveness and economic assessment of routine larviciding for prevention of chikungunya and dengue in temperate urban settings in Europe

G. Guzzetta [a], F. Trentini [b], P. Poletti [a, b], F. Baldacchino [c], F. Montarsi [d], G. Capelli [d], A. Rizzoli [c], R. Rosa' [c], S. Merler [a], A. Melegaro [b, e]

a: Fondazione Bruno Kessler, Trento, Italy

b: Dondena Centre for Research on Social Dynamics and Public Policy, Bocconi University, Milan, Italy.

c: Department of Biodiversity and Molecular Ecology, Research and Innovation Centre, Fondazione Edmund Mach, San Michele all'Adige (Trento), Italy

d: Istituto Zooprofilattico Sperimentale delle Venezie, Padova, Italy

e: Department of Policy Analysis and Public Management, Bocconi University, Milan

1. Additional information on input data

Figure S1.1 shows the location of the 70 trapping sites distributed over 10 municipalities in the provinces of Trento and Belluno; the location of the study area with respect to the map of Italy is shown in the inset. Figure S1.2 shows the daily average temperatures measured in the 10 municipalities for the two mosquito seasons; the mean over different traps from the same municipality is reported. Between mid-June and September, temperatures in 2015 were constantly higher than corresponding ones registered in the same dates in 2014. The figure shows that temperature variability across sites is smaller than inter-year variability.

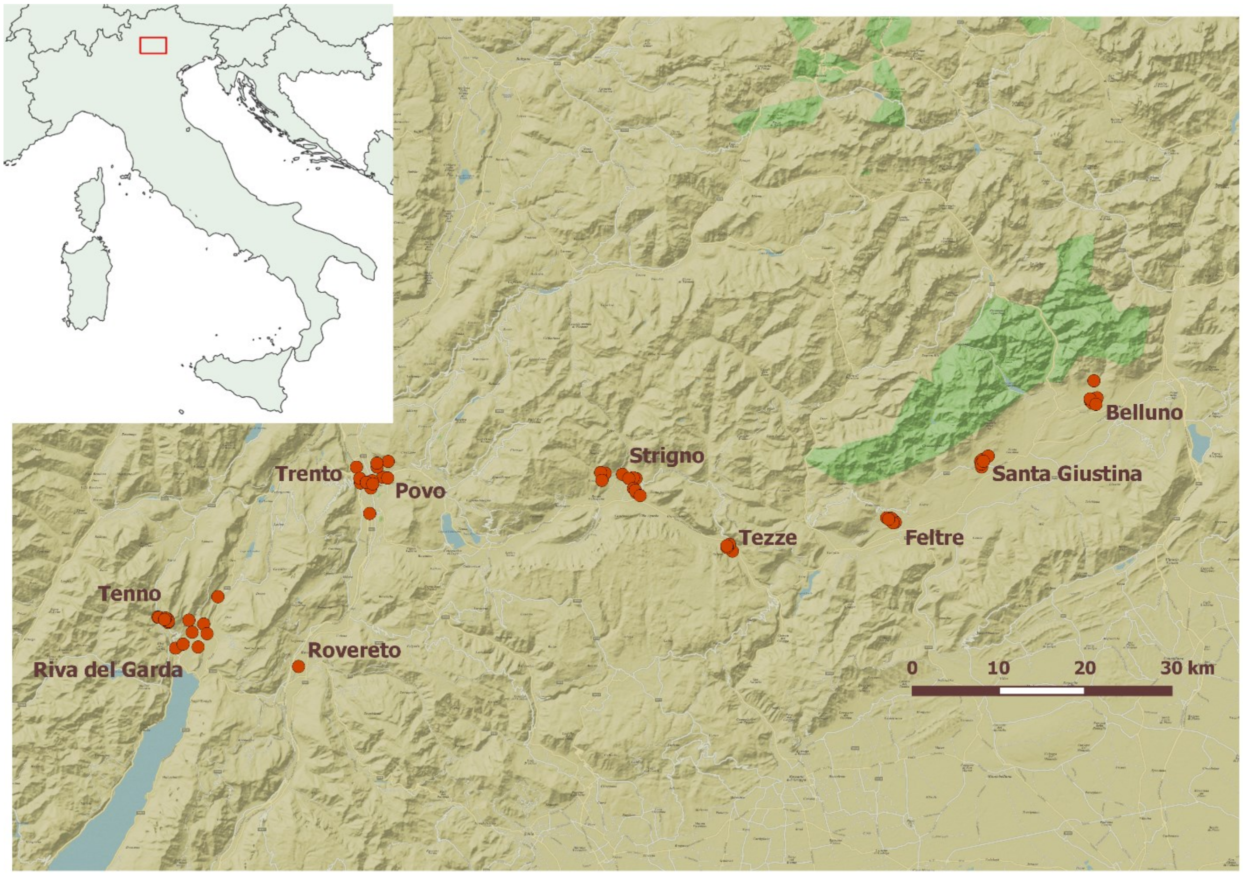

Figure S1.1. *Map of trapping locations and corresponding municipalities. Inset: location in Italy of the study area.*

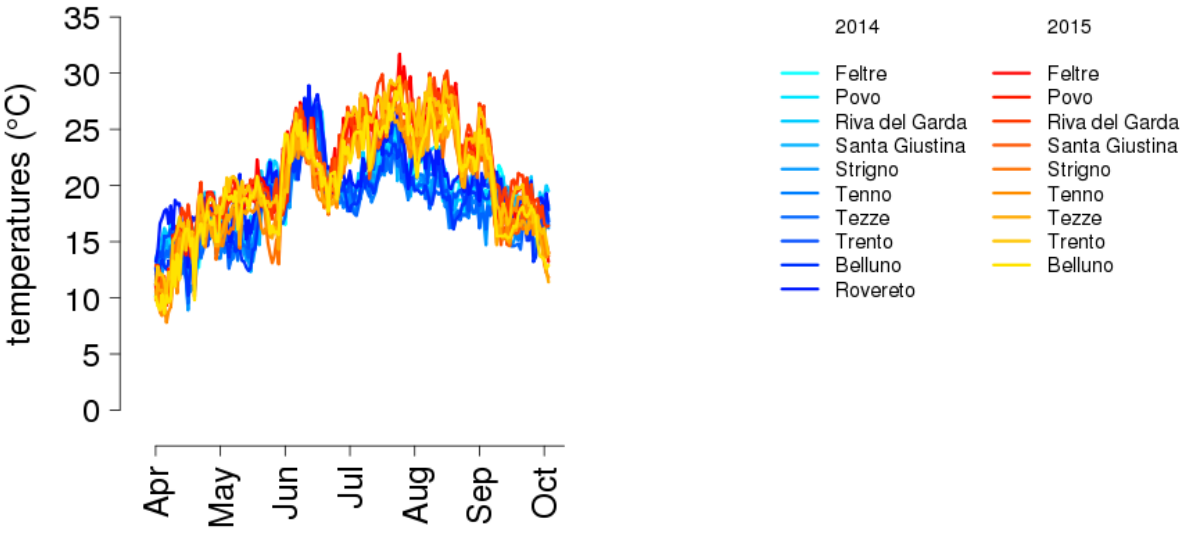

Figure S1.2. *Temperatures over time at each site and for the two study years.*

2. Mosquito population model

Equations for the mosquito population model are taken from [S1] and reported below.

$$\begin{cases} \dot{E} = n_E g_V V - (m_E + d_E)\, E \\ \dot{L} = d_E E - \left(m_L \left(1 + \frac{L}{a_{sy}}\right) + d_L\right) L \\ \dot{P} = d_L L - (m_P + d_P) P \\ \dot{V} = \frac{1}{2} d_P P - (m_V + \alpha(t))\, V \end{cases} \quad \text{(Eq S1)}$$

E, L, P and V represent populations in the four developmental stages of mosquitoes, i.e. eggs, larvae, pupae and adult mosquitoes respectively. Fixed model parameters are the stage-specific mortality (*m*) and developmental rates (*d*) from one stage to the next; g_V, whose inverse represents the gonotrophic cycle; and the number of eggs per oviposition n_E; all fixed parameters are temperature-dependent according to functions described in [S2], except for n_E, which is set to 60 [S2]. Free model parameters are the capture rate α, which is different from zero only in days where traps are active (hence the dependence on time *t* in the equation); and the coefficients coding density-dependence for larval mortality, a_{sy}, which vary by site *s* and year *y*, and represent a measure of the habitat suitability. Parameters were calibrated to reproduce capture data according to an MCMC procedure based on the Poisson likelihood of captures, as described fully in [S1]. For each site, year, disease, intervention scenario and coverage value, 100 sets of parameter values were sampled from the posterior distributions of the calibrated mosquito population model; to account for model stochasticity, 100 random repetitions were run for each parameter set.

Table S1 reports the mean and 95% confidence interval of the posterior distribution of free parameters, while Figure S3.1 shows a comparison between observed (black dots) and model-predicted (red dots, with 95% confidence interval) captures at all sites and in the two years. The R^2 computed between observed and model-predicted values is 0.77.

Site	Habitat suitability parameter				Capture rate (%/day)	
	2014		2015		Mean	95%CI
	Mean	95%CI	Mean	95%CI		
Feltre	44.5	41.4-49.1	32.6	29.8-34.9	1.64	1.53-1.73
Povo	27.5	24.8-30.0	31.9	28.1-35.1		
Riva del Garda	30.0	26.6-32.6	33.6	30.7-36.0		
Santa Giustina	34.5	30.3-38.6	25.2	23.3-27.3		
Strigno	3.7	3.1-4.4	3.2	3.0-3.7		
Tenno	15.5	12.4-19.6	32.0	29.6-34.2		
Tezze	25.2	22.2-28.2	28.8	26.3-31.9		
Trento	24.9	22.1-27.4	25.8	23.6-28.2		
Belluno	6.5	4.5-8.7	7.9	7.0-9.0		
Rovereto	22.9	17.4-30.2	-	-		

Table S1. *Estimated site- and year- specific habitat suitability and capture rate parameters*

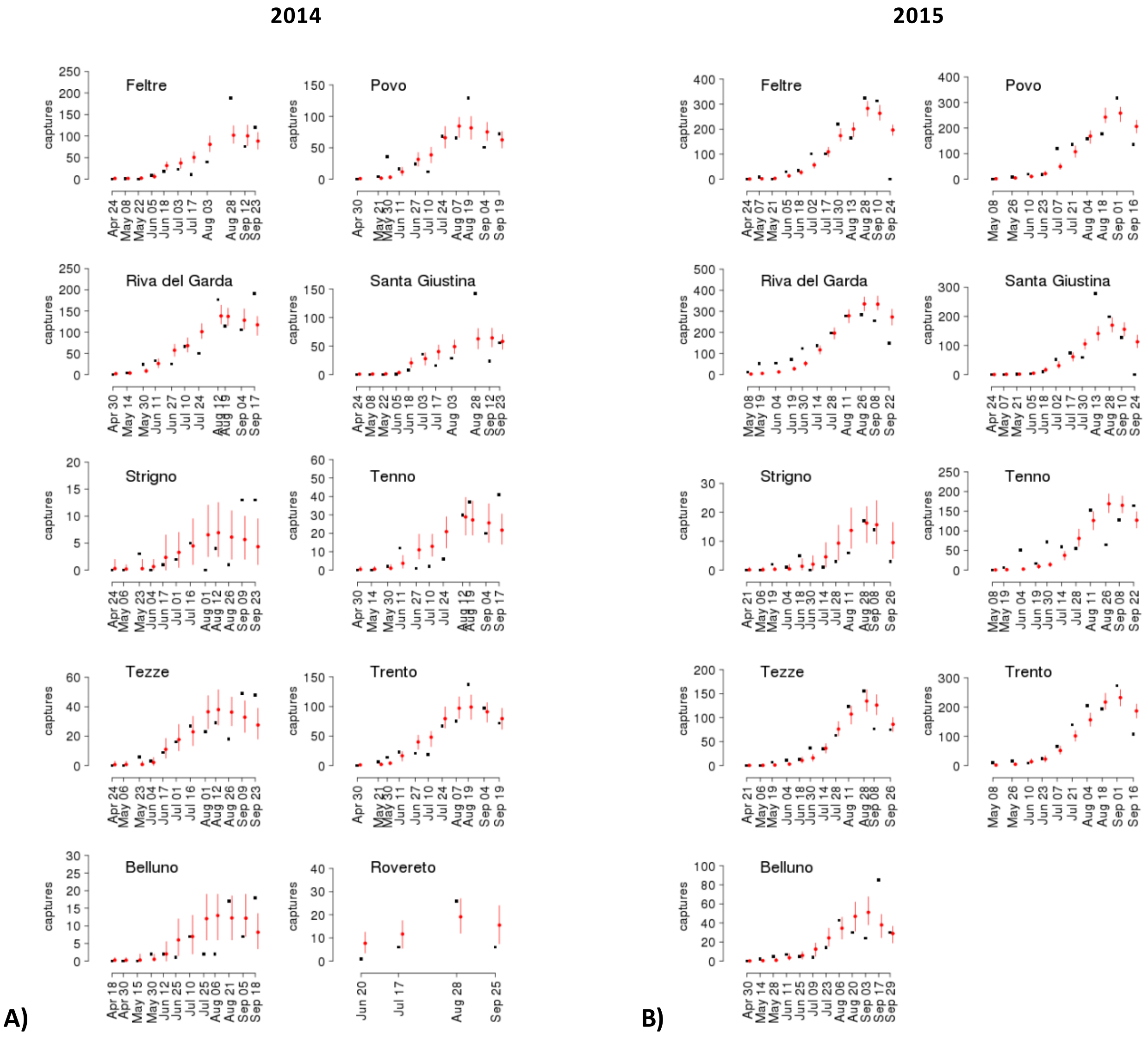

Figure S2.1. *Comparison between observed and model-predicted captures in each site; A) 2014; B) 2015.*

In Figure S2.2 we show model-predicted densities of adult female mosquitoes in the absence of control interventions for the two seasons and 10 study sites.

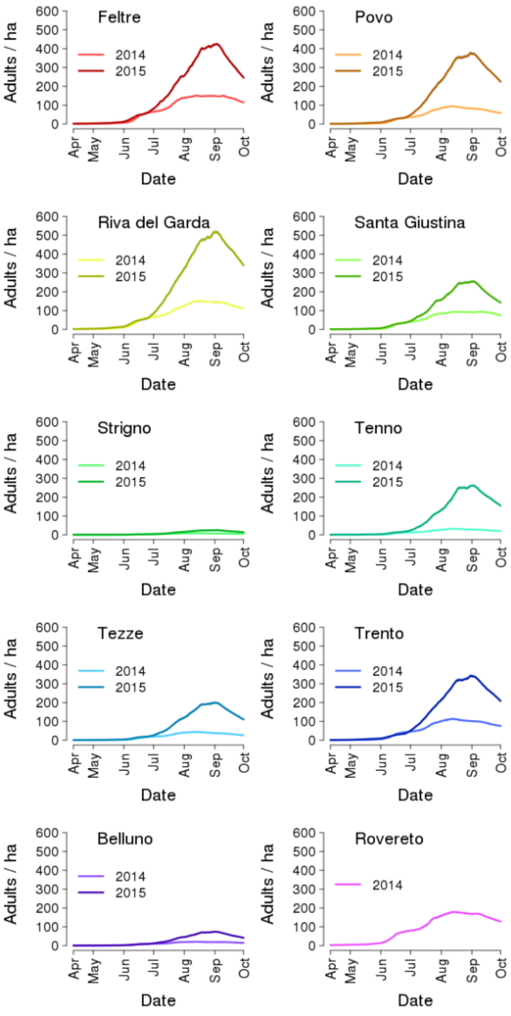

Figure S2.2. *Model-predicted adult density by site and year.*

3. Modeling larvicides

The population model was modified to include larvicide interventions. We denote by c the intervention coverage, i.e. the proportion of all breeding sites on which larvicide treatment is actually performed. We assume that aquatic stages (eggs, larvae and pupae) are equally distributed across treated and untreated catch basins so that, for example, $E_{treated} = c\,E$ and $E_{untreated} = (1-c)\,E$ and similarly for larvae and pupae. Larvicidal treatment is assumed to instantaneously kill existing larvae, therefore the total larval population just after treatment, $L(T_+)$, will be given by

$$L(T_+) = (1 - c)\,L(T_-) \qquad \text{(Eq. S2)}$$

where T_- and T_+ are, respectively, the times immediately before and immediately after initiation of the treatment intervention. For the duration of treatment, eggs hatching in treated catch basins are assumed to die without developing into larvae; furthermore, the density-dependent mortality parameters is reduced accordingly, in order to account for the decrease in the number of viable breeding sites. Therefore, for the duration of treatment only, the equation regulating the dynamics of larvae is represented by Equation S3.

$$\dot{L} = d_E\,(1-c)E - \left(m_L\left(1+\frac{L}{(1-c)a_{sy}}\right)+d_L\right)L \qquad \text{(Eq. S3)}$$

For each type of intervention (public only or supplemented by the involvement of private citizens), we assessed larvicide effectiveness under two coverage values representing a realistic range. In general, coverage can be expressed as:

$$c = \frac{q_{pub}\,b_{pub}+q_{priv}b_{priv}}{b_{pub}+b_{priv}} \qquad \text{(Eq. S4)}$$

where *q* is the fraction of (public or private) catch basins which are effectively treated and *b* is the total number of existing (public or private) breeding sites. Equivalently, *b* can be expressed in terms of the breeding site density per unit area. In a large-scale survey of different types of breeding sites conducted in urban areas in northern Italy [S3], it was found that 94% of the pupal population was produced within catch basins, while other types of water-filled containers (such as plant saucers, drums and buckets) contributed marginally to the abundance of adult mosquitoes. Based on these findings, we approximated the number of breeding sites in a given area with the number of catch basins; furthermore, we did not consider the effect of control interventions directed to the removal of other water-filled containers. Vector control interventions by municipalities can be designed to cover all public catch basins; however, some catch basins may be missed or treatment may be ineffective for various reasons, e.g. flushing, dilution or rapid dissolution of the larvicide product: therefore, we assumed that q_{pub} is between 85% and 95%. Results from a pilot study on the involvement of citizens in mosquito control from San Michele all'Adige, a small town in the province of Trento, suggest a value for q_{priv} between 45% and 55% (F. Baldacchino, personal communication). The much lower coverage of private interventions depends on several issues, including the presence of abandoned premises, difficulties in contacting reference persons, occasional denial of collaboration, and the actual compliance of citizens nominally adhering to the program. For what concerns the density of catch basins, we base our estimates on a survey conducted in San Michele all'Adige within the above-mentioned pilot study, which found b_{pub} = 16.8 per hectare and b_{priv} around 30.7 per hectare (F. Baldacchino, personal communication). These results are consistent with a previous survey in northern Italy [S3], which estimated b_{pub} between 7 and 19 per hectare and b_{priv} at about 36.3 per hectare. With the given estimates for *q* and *b*, we obtain a range for realistic coverage values of about 30% to 50% for public interventions (where $q_{priv}=0$), and 60% to 75% for interventions including private premises.

We considered 24 intervention scenarios, which differ by the number of treatments (effort level) within a mosquito season and by starting date. We considered between 1 and 4 treatments within a season, and we assume that each re-treatment is performed 30 days after the last treatment, so that the effect of larvicide is kept constant throughout the intervention. Possible starting dates were sampled at intervals of 15 days between the 1st of May and the 1st of September, and we considered only scenarios whose overall effectiveness end before October 1st (Figure S3.1). In this way, we obtain 9 scenarios with single interventions, 7 with two treatments, 5 with three treatments and 3 with four treatments, the latter covering almost the whole mosquito season. For interventions with involvement of private

citizens, we assume for simplicity that treatments in private premises are perfectly synchronized with public ones.

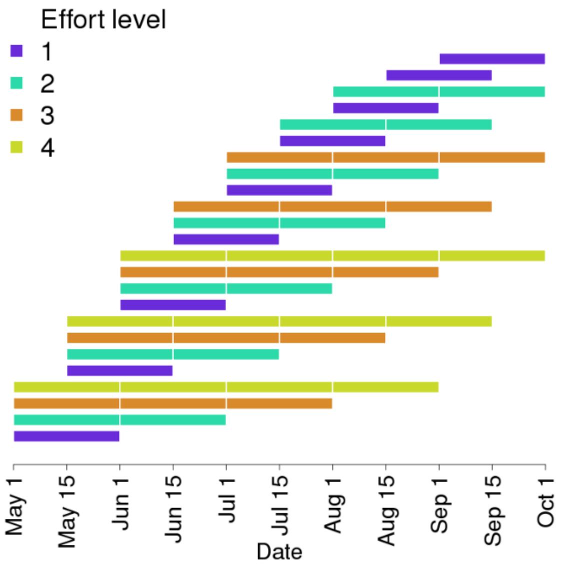

Figure S3.1. *Scenarios for larvicide intervention considered in our analysis. Horizontal bars indicate the 30 days window of effectiveness of a single intervention. Re-treatments are performed after 30 days since the start of the last treatment.*

Figures S3.2 reports the resulting average density of female mosquitoes in the different municipalities and years by different effort levels of public larviciding, under optimally timed interventions and for the two values of coverage. Figure S3.3 reports analogous numbers for the public and private intervention.

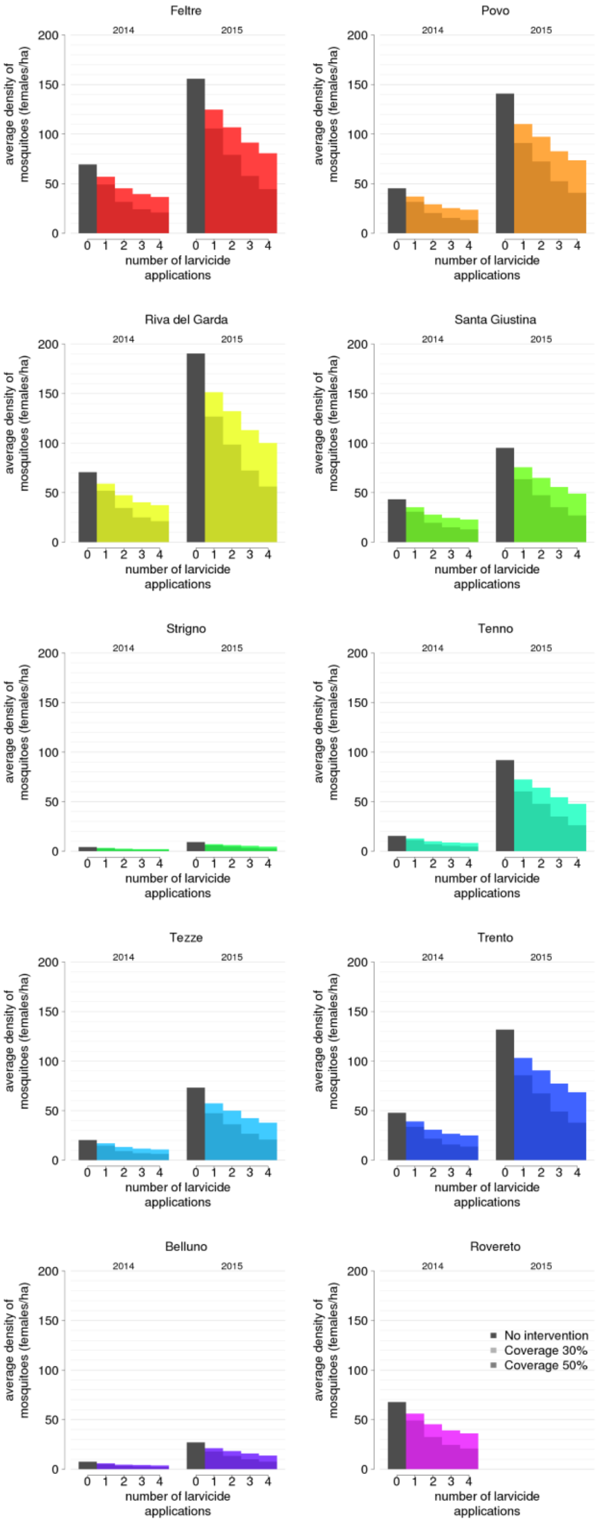

Figure S3.2. *Expected density of female mosquitoes with optimally timed interventions in public breeding sites, for different coverages and effort levels, disaggregated by site and year.*

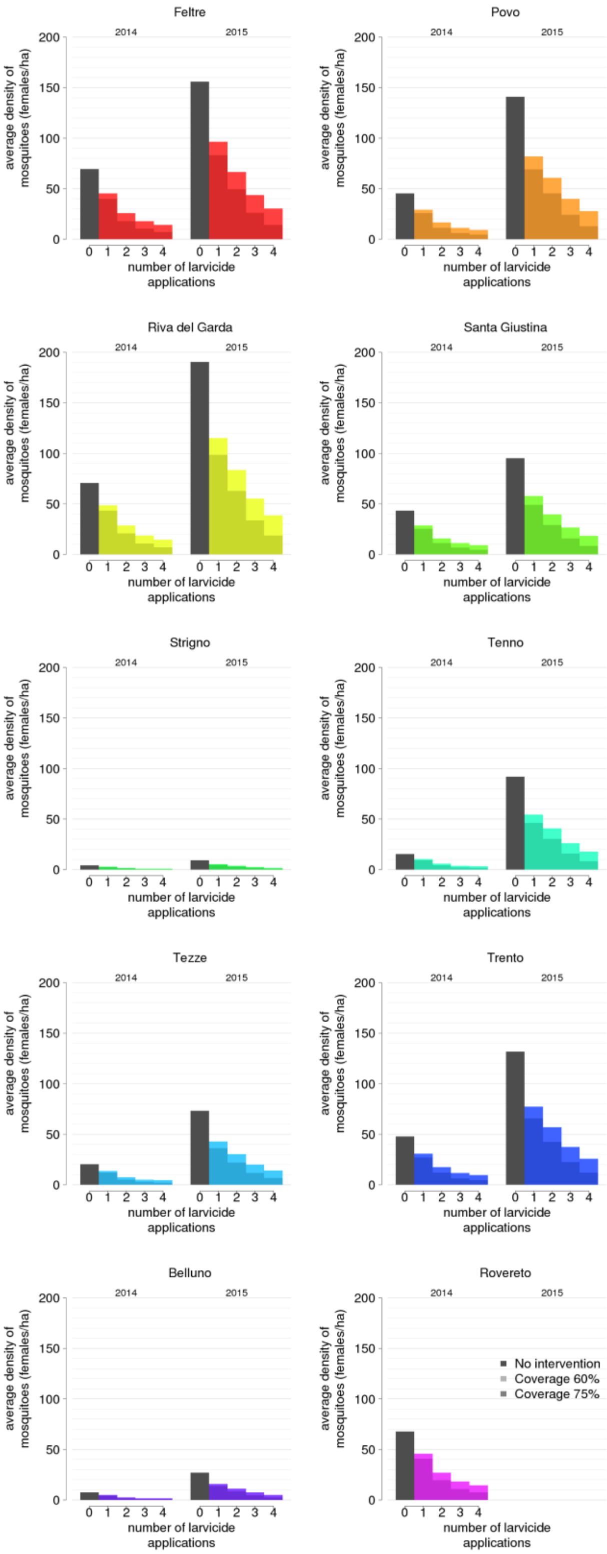

Figure S3.3. *Expected density of female mosquitoes with optimally timed interventions in public and private breeding sites, for different coverages and effort levels, disaggregated by site and year.*

4. Transmission dynamic model

The adopted transmission dynamic model is a standard SEI-SEIR model [S2], which can be mathematically expressed as Equations S5:

$$\begin{cases} \dot{V_S} = \frac{1}{2} d_P P - (m_V + \lambda_V)\, V_S \\ \dot{V_E} = -m_V V_E + \lambda_V V_S - \omega_V V_E \\ \dot{V_I} = -m_V V_I + \omega_V V_E \\ \dot{H_S} = -\lambda_H H_S \\ \dot{H_E} = \lambda_H H_S - \omega_H H_E \\ \dot{H_I} = \omega_H H_E - \gamma H_I \\ \dot{H_R} = \gamma H_I \end{cases} \qquad \text{(Eq. S5)}$$

where V_S, V_E and V_I are the number of vectors in different infection states (susceptible, exposed and infected respectively). We assume that vectors from all infection states have the same temperature-dependent mortality rate m_V and that new female adults (given by the term ½ d_p P) begin their adult life as susceptible (i.e. no vertical transmission in mosquitoes). Mosquitoes may become infected through blood meals at a rate λ_V, become infectious after an average time called "extrinsic incubation period" and given by $1/\omega_V$. Infectious mosquitoes remain so throughout the rest of their life. The force of infection on mosquitoes λ_V is given by Equation S6:

$$\lambda_V = k\, \chi_V \frac{H_I}{N} \qquad \text{(Eq S6)}$$

where k is the mosquito biting rate, χ_V is the probability that a mosquito becomes infected upon a single blood meal on an infectious human, H_I is the number of infectious humans and N is the total human population ($N=H_S+H_E+H_I+H_R$). H_S, H_E, and H_R represent, respectively, the remaining infection states for humans, namely susceptible, exposed and recovered. Humans may acquire infection with a rate λ_H, become infectious after an average time called "intrinsic incubation period" given by $1/\omega_H$, and become lifelong immune after recovery with a rate γ. The force of infection on humans λ_H is given by Equation S7

$$\lambda_H = k\, \chi_H \frac{V_I}{N} \qquad \text{(Eq S7)}$$

where χ_H is the probability that a human becomes infected upon a single bite from an infectious mosquito.

In a previous study on a Chikungunya outbreak in northern Italy, the biting rate *k* was estimated at 0.09 bites per adult mosquito per day [S2]. Parameter values for the two diseases were also obtained from previously published modeling studies; they are reported in Table S2.

		Chikungunya		Dengue	
	**Unit**	**Value**	**Reference**	**Value**	**Reference**
χ_V	%	77	[S2]	22-42*	[S4]
χ_H	%	70	[S2]	26-44*	[S4]
$1/\gamma$	days	4.5	[S2]	4	[S5]
$1/\omega_V$	days	2.5	[S2]	2.4-4.6*	[S4]
$1/\omega_H$	days	3	[S2]	2	[S5]

Table S2. *Epidemiological parameters for chikungunya and dengue.*

**: temperature-dependent parameters (see Equations S8-S10); we report the range across sites and years of average values computed between May 1st and September 30.*

For dengue, some epidemiological parameters are temperature-dependent according to Equations S8-S10 [S4]:

$$\chi_V = 0.0729\,T - 0.97 \quad \text{(Eq S8)}$$

$$\chi_H = 0.001044\,T\,(T - 12.286)\,\sqrt{32.461 - T} \quad \text{(Eq S9)}$$

$$\omega_V = \frac{0.003359 \frac{T_k}{298} e^{\frac{15000}{R}\left(\frac{1}{298} - \frac{1}{T_k}\right)}}{1 + e^{\left(\frac{6.023\,10^{21}}{R}\left(\frac{1}{-2.176\,10^{30}} - \frac{1}{T_k}\right)\right)}} \quad \text{(Eq S10)}$$

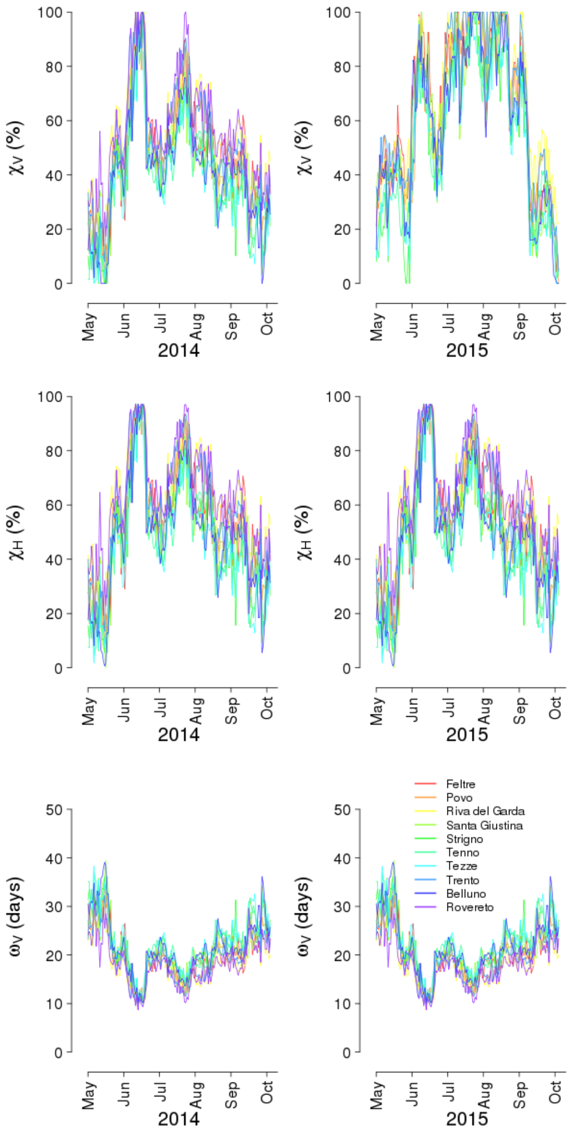

Figure S4.1. *Temperature-dependent epidemiological parameters for dengue over time in the two study years.*

where T is the temperature in Celsius degrees, T_k is the temperature in Kelvin degrees and R is Avogadro's constant. Resulting seasonal averages of parameter values are reported in Table S2, while actual temporal values over the two considered mosquito seasons are shown in Figure S4.1.

5. Material and methods for the economic analysis

The average cost per case and DALY loss per case were derived using a decision tree approach and considering the probabilities associated to both a dengue and a chikungunya case of being symptomatic and asymptomatic, severe and non-severe and the various disease outcomes (death, hospitalization and ambulatory assistance) (Figure S5.1).

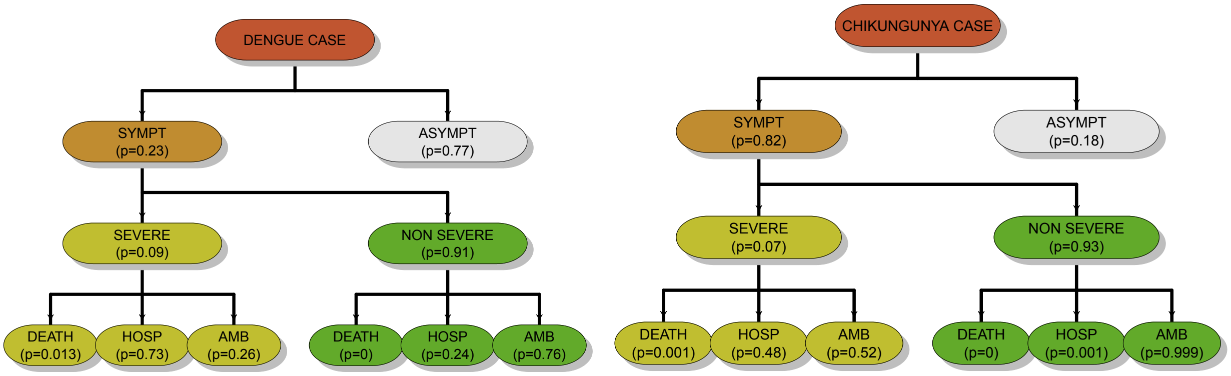

Figure S5.1. *Decision trees for the classification of cases of Dengue and Chikungunya*

In particular, the average cost per case and DALY loss per case were derived as in the following and considering base case parameters' values as indicated in Tables S5.1 and S5.2:

$$COST = p_{sym}[p_{sev}(p_{amb} + p_{death})(n_{visit}cost_{visit} + cost_{treat\_amb}) + p_{sev}(p_{hosp} + p_{death})(n_{days\_sev}cost_{hosp\_stay} + cost_{treat\_hosp}) + p_{no\_sev}p_{amb}(n_{visit}cost_{visit} + cost_{treat\_amb}) + p_{no\_sev}p_{hosp}(n_{days\_no\_sev}cost_{hosp\_stay} + cost_{treat\_hosp})$$

$$DALY = p_{death}(YYL + dw_{sev}n_{ill\_day\_sev}) + p_{sev}(dw_{sev}n_{ill\_day\_sev}) + p_{no\_sev}(dw_{no\_sev}n_{ill\_day\_no\_sev})$$

In Tables S5.1 and S5.2 also probability distributions of economic model parameters are shown. These were used to incorporate the existing uncertainties in the probabilistic sensitivity analysis. Cost parameters were derived from analyses of national Hospital Discharge System (SDO) data and from the expert opinion provided by doctors from the Department of Infectious Diseases of the San Matteo hospital in Pavia, Italy. These costs are based on an average of 2 ambulatory visits per patient, with a general practitioner (GP) cost of 47.5 euros (range 45-50) and tests/treatment costs of 328.4 euros (250-407). The cost of a hospitalized patient was derived by multiplying the cost per day of hospitalization of 391.7 euros (370-413) to the estimated average inpatient stay and adding the average cost for tests/treatments within the hospital, consisting of 1534.5 euros per case (1400-1670).

Model input parameter	Value (range)	Distribution for PSA	Source
Epidemiological parameters - Dengue			
Proportion of symptomatic cases - p_{sym}	.23 (.07, .47)	TruncNorm (.23, .002)	[S6]
Proportion of severe cases (SV) - p_{sev}	.09 (.06, .13)	Beta (76.48, 756.41)	Own calculation based on SDO
Proportion of hospitalization (SV) - p_{hosp}	.73 (.52, .88)	Beta (56.97, 21.36)	Own calculation based on SDO
Proportion of hospitalization (non SV) - $p_{nosev}*p_{hosp}$	.24 (.09, .46)	Beta (15, 48)	[S7]
Proportion of deaths (SV) - p_{death}	.013 (.001, .04)	Beta (6.66, 505.58)	[S8]
Cost of illness - Dengue			
Length of hospital stay in days (SV) - n_{day_sev}	5.3 (2.2, 10.46)	Gamma(23.21, .23)	Own calculation based on SDO
Length of hospital stay in days (non SV) - $n_{day\_no\_sev}$	3.8 (1.6, 7.24)	Gamma(23.21, .16)	[S9]
Number of ambulatory visits (both) - n_{visit}	2	Point estimate	Expert opinion
Cost per ambulatory visit - $cost_{visit}$	47.5 (40, 55)	Triangular	Expert opinion
Treatment and test cost for an ambulatory case - $cost_{treat\_amb}$	328.4 (250, 406.8)	Triangular	Expert opinion
Treatment and test cost for an hospitalized case - $cost_{treat\_hosp}$	1522.97 (1400, 1645.94)	Triangular	Expert opinion
Hospital stay cost per day - $cost_{hosp\_stay}$	391.7 (370, 413.4)	Triangular	Expert opinion
Burden of disease - Dengue			
Duration of illness in days (SV) - $n_{ill\_day\_sev}$	8.31 (3.96, 14.3)	Gamma(30.69, .27)	Own calculation based on [S10]
Duration of illness in days (non SV) - $n_{ill\_day\_no\_sev}$	4.36 (1.88, 4.37)	Gamma(30.69, .14)	Own calculation based on [S10]
Disability weights for a severe case - dw_{sev}	.545 (.47, .62)	Beta (337.32, 281.62)	[S8]
Disability weights for a non severe case - dw_{no_sev}	.197 (.16, .24)	Beta (313.38, 1271.76)	[S8]
Years life lost in case of death - *YLL*	38 (32, 44)	Uniform	[S11]

Table S5.1. *Distributions of parameters of the economic model for Dengue*

Model input parameter	Value (range)	Distribution for PSA	Source
Epidemiological parameters - Chikungunya			
Proportion of symptomatic cases - p_{sym}	.82 (.7, .9)	TruncNorm (.82, .02)	[S12]
Proportion of severe cases (SV) - p_{sev}	.07 (.001, .296)	Beta (3.69, 48.76)	Own calculation based on SDO
Proportion of hospitalization (SV) - p_{hosp}	.48 (.26, .68)	Beta (47.32,51.5)	Own calculation based on SDO
Proportion of hospitalization (non SV) - $p_{nosev}*p_{hosp}$	.001 (.000, .01)	Beta (0.99,997.2)	Own calculation based on SDO
Proportion of deaths (SV) - p_{death}	.001 (.000, .001)	Beta (0.99,9997)	[S13]
Cost of illness - Chikungunya			
Length of hospital stay in days (SV) - n_{day_sev}	4.17 (2, 7.9)	Gamma(30.86, .135)	Own calculation based on SDO
Length of hospital stay in days (non SV) - $n_{day\_no\_sev}$	3.8 (1.8, 3.8)	Gamma(34.18, .11)	Own calculation based on SDO
Number of ambulatory visits (both) - n_{visit}	2	Point estimate	Expert opinion
Cost per ambulatory visit - $cost_{visit}$	47.5 (40, 55)	Triangular	Expert opinion
Treatment and test cost for an ambulatory case - $cost_{treat\_amb}$	328.4 (250, 406.8)	Triangular	Expert opinion
Treatment and test cost for an hospitalized case - $cost_{treat\_hosp}$	1534 .5(1400, 1670)	Triangular	Expert opinion
Hospital stay cost per day - $cost_{hosp\_stay}$	391.7 (370, 413.4)	Triangular	Expert opinion
Burden of disease - Chikungunya			
Duration of illness in days (SV) - $n_{ill\_day\_sev}$	3.2 (0.07, 15.13)	Gamma(3.14, 1.02)	Own calculation based on SDO
Duration of illness in days (non SV) - $n_{ill\_day\_no\_sev}$	2.9 (0.05, 13.75)	Gamma(2.58, 1.13)	Own calculation based on SDO
Disability weights for a severe case - dw_{sev}	.428 (.38, .47)	Beta (1047.4, 1399.7)	[S13]
Disability weights for a non severe case - dw_{no_sev}	.195 (.16, .24)	Beta (305.9, 1262.84)	[S13]
Years life lost in case of death - *YLL*	20 (0, 40)	Uniform	[S13]

Table S5.2. *Distributions of parameters of the economic model for Chikungunya*

The costs sustained for the two compared strategies (public vs private larvicidal applications) were obtained according to the parameters shown in Table S5.3. Data in Tab. S5.3 were obtained from a pilot study designed jointly by Fondazione Edmund Mach (FEM) and Istituto Zooprofilattico Sperimentale delle Venezie (IZSVE) [S14].

Costs such as those of surveillance activities, public sanitation and public education are fixed costs, and are considered for both private and public interventions.

$$COST_{surv} = cost_{ovitraps} + cost_{surv\_pers}$$

$$COST_{edu} = n_{inh}(cost_{meeting\_per\_inh} + cost_{broch\_per\_inh})$$

$$COST_{sanit} = cost_{sanit\_pers}$$

The final cost, respectively per catch basin and per household, for the public and the private interventions are computed as follows

$$COST_{cb\_pub} = n_{cb}(cost_{larv\_prod} + cost_{larv\_pers} + cost_{larv\_mapp})$$

$$COST_{home\_priv} = n_{tab\_per\_home} cost_{larv\_prod} + n_{hours\_per\_home} cost_{pers}$$

In particular, the cost of larvicide treatments on public spaces was estimated to be around 1.17 euros per catch basin (0.80-1.70), including costs for both personnel and larvicide products. The cost for door-to-door interventions was found to be 12.66 euros per premise (4.80-30.12), including both personnel costs for home visits and costs of larvicide products. To these, we added 0.95 euros per inhabitant (CI 0.46-1.43), for the organization of an education campaign made of public meetings for residents to explain the benefits of larvicide applications and facilitate the acceptability of the intervention in private premises [S14].

Cost of Intervention parameters			
Total cost ovitraps	219.13 (93.2, 344.75)	Triangular	Own assumption based on IZSVE and FEM data
Personnel cost	1655.32 (912.8, 2400)	Triangular	Own assumption based on IZSVE and FEM data
Cost of larvicidal product* n. 1 (per catch basin) - $cost_{larv\_prod}$	.147	Point Estimate	Own assumption based on IZSVE and FEM data
Personnel cost mapping (per catch basin) - $cost_{larv\_mapp}$	.41 (.35, .46)	Triangular	Own calculation based on IZSVE and FEM data
Personnel cost treatment (per catch basin) - $cost_{larv\_pers}$	.62 (.27, 1.1)	Triangular	Own calculation based on IZSVE and FEM data
Cost of larvicidal product* n.2 (per tab) - $cost_{larv\_prod}$	.65	Point Estimate	Own assumption based on IZSVE and FEM data
Number of tabs (per household) - $n_{tab\_per\_home}$	.69 (.04, 2.49)	Gamma(30.03, .144)	Own calculation based on IZSVE and FEM data
Personnel cost (per household) - $cost_{pers}$	15.25 (14.76, 16)	Triangular	Own calculation based on IZSVE and FEM data
Number of working hours (per household) - $n_{hours\_per\_home}$	.64 (.16, 1.52)	Gamma(13.9,3 0.05)	Own calculation based on IZSVE and FEM data
Personnel cost per public meetings (per inh)	.28 (.11, .46)	Triangular	Own calculation based on IZSVE and FEM data
Cost per brochure (per inh)	.68 (.35, .99)	Triangular	Own calculation based on IZSVE and FEM data
Personnel cost per public sanitation (per ha)	9.02 (8, 10)	Triangular	Own calculation based on IZSVE and FEM data

Table S5.3. *Distributions of parameters for the cost of interventions*

** The reference product is Bacillus thuringiensis var. israelensis + Bacillus sphaericus*

The net health benefit (NHB) was derived for each intervention and uncertainty around its average value was taken into consideration through simulations from the parameters distributions presented above.

$$NHB = \Delta D - \Delta C/k$$

where ΔD and ΔD are respectively DALY averted and incremental costs due to intervention, and the willingness to pay (WTP) k, as mentioned in the main text, was fixed at 35.000 euros. Such value can be interpreted as the amount of money the public Italian healthcare system is willing to pay for each DALY averted.

For each intervention scenario and each site, stochastic realizations of the NHB were drawn according to the distributions of the DALY loss per case and cost per case shown in Fig S5.2 and to the distribution of intervention costs shown in Fig. S5.3.

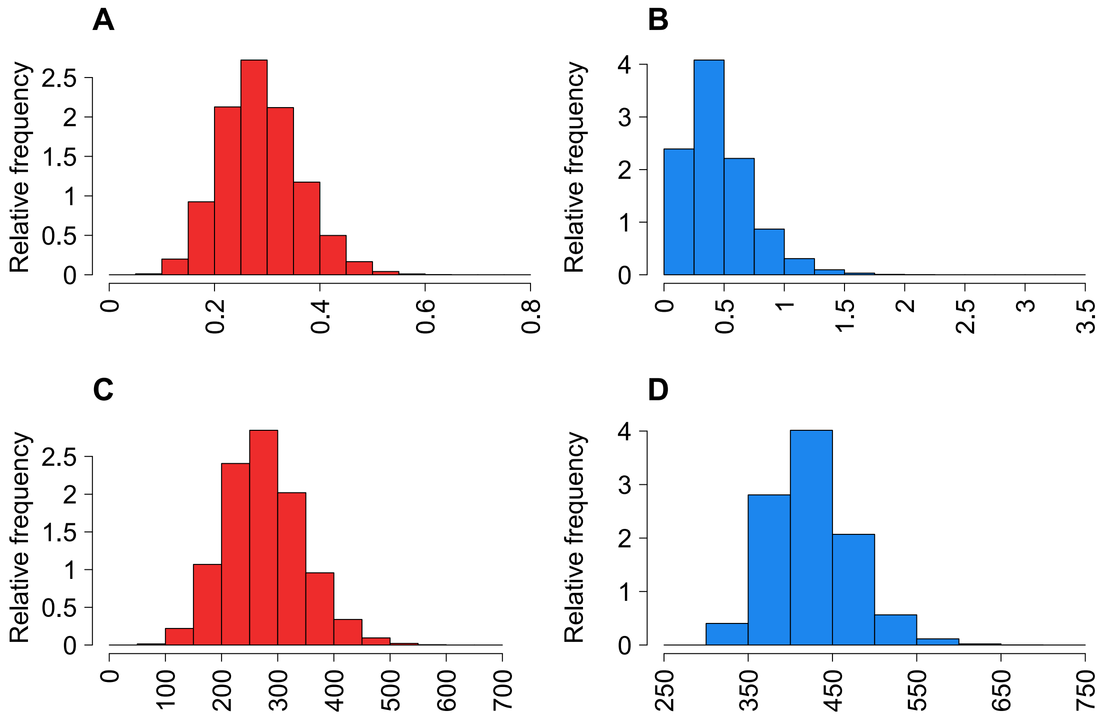

Figure S5.2. *Distributions of DALY loss and cost per case for Dengue (respectively panel A and C) and for Chikungunya (B and D)*

Optimal strategies are associated with the highest NHB, therefore probabilities in Fig. 3 and Fig. 4 of the main text are computed as the fraction of simulations for which each intervention has the highest net health benefit.

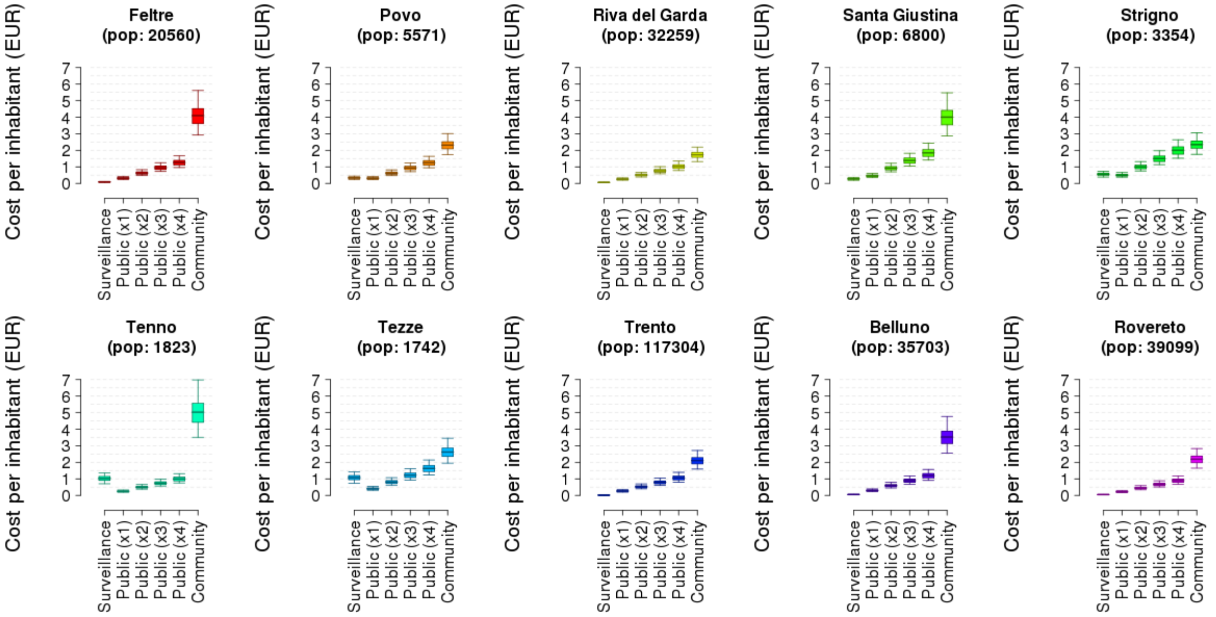

Figure S5.3. *Distributions of costs per inhabitant of different interventions at each site. Boxplots represent the 95% confidence interval, interquantile ranges and mean costs. "Community" refers to interventions involving inhabitants in larviciding activities within private premises.*

6. Questionnaire on budgeted and sustained cost for control programs against *Ae. albopictus* in Trentino Alto-Adige in 2013

A questionnaire was designed to assess the vector control policies of different Municipalities in Trentino Alto Adige in a 5-year time frame, from 2009 to 2013. We collected answers from 77 municipalities, among which 17 reported the presence of *Ae. albopictus* and 12 implemented one or more control interventions.

Information were collected on the general characteristics of the municipalities, budget allocated for the control of *Ae. Albopictus*, activities implemented during the five years (among which surveillance, larvicidal etc), the level of coordination with the Province and the Region, awareness campaigns (if any), and related expenses for each implemented activity. Eight Municipalities responded thoroughly to the questionnaire. According to their answers, the average budget and expenses for activities implemented in order to control the spread of Ae. Albopictus were respectively 0.714 (0.254, 1.192) and 0.611 (0.016,1.077) euros per inhabitant, see Tab S6.1 for details. These values were found to be in line with estimates obtained for the vector control programs implemented in Emilia Romagna after the 2007 outbreak.

MUNICIPALITY	AWARENESS	SURVEILLANCE	LARVICIDAL	ADULTICIDE	EXPENSE (euros per inhabitant)
ALDENO	X		X		0.88
ARCO	X	X	X		0.35

AVIO	X	X	X	X	0.85
BESENELLO	X	X	X		1.08
COMUNITA' ALTO GARDA E LEDRO *	X	X	X		0.33
NAGO TORBOLE	X	X	X	X	1.05
RIVA DEL GARDA	X	X	X		0.53
TRENTO	X	X	X		0.13
VOLANO	X	X	X	X	0.02

Table S6.1. *Implemented interventions and related average expense per inhabitant for municipalities in the province of Trento in 2013*

** Comunità Alto Garda e Ledro appears as a single record in the questionnaire but it involves different municipalities, including Riva del Garda and Arco. It is reported for informational purposes but it is not considered in the analysis*

7. Effect of larviciding on dengue

Figures S7.1-S7.3 show model results on the epidemiological effectiveness of larviciding in reducing potential dengue transmission (as in Figures 2B and 3 in the main text for chikungunya). In particular, Figure S7.1 shows the expected number of secondary cases, Figure S7.2 the probability of local transmission and Figure S7.3 the outbreak size distributions.

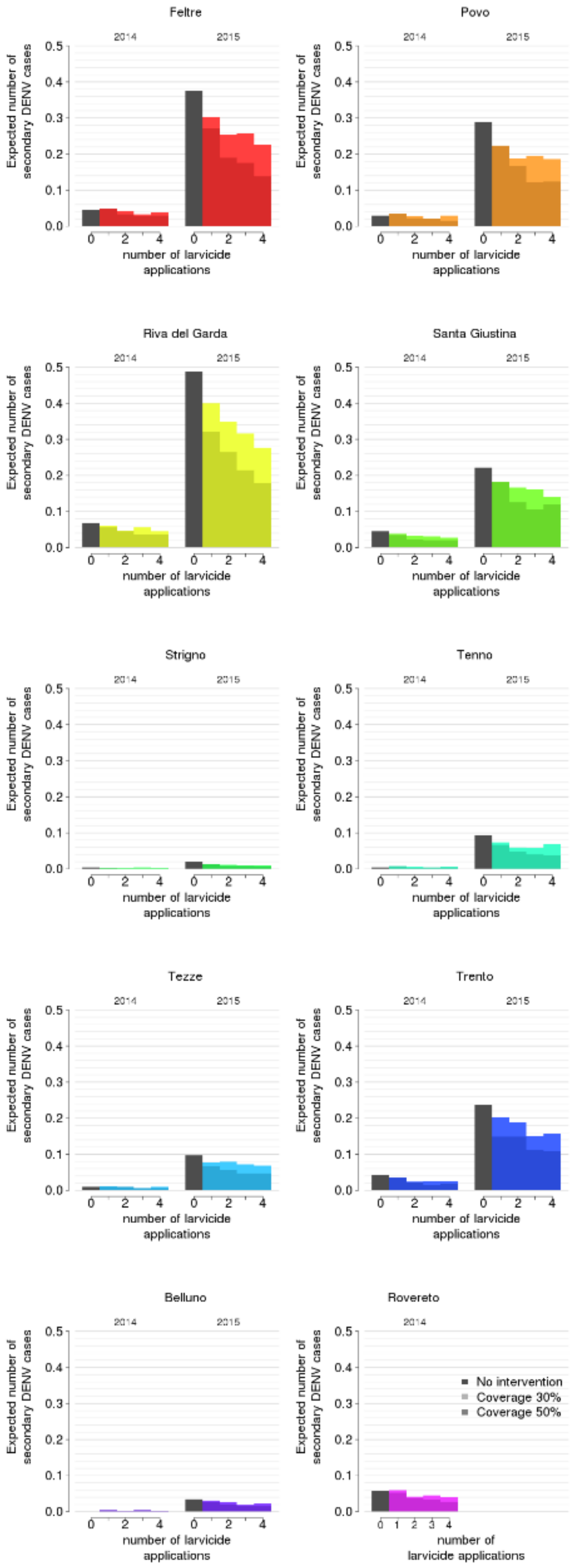

Figure S7.1. *Expected number of dengue cases with optimally timed interventions, for different coverages and effort levels, disaggregated by site and year.*

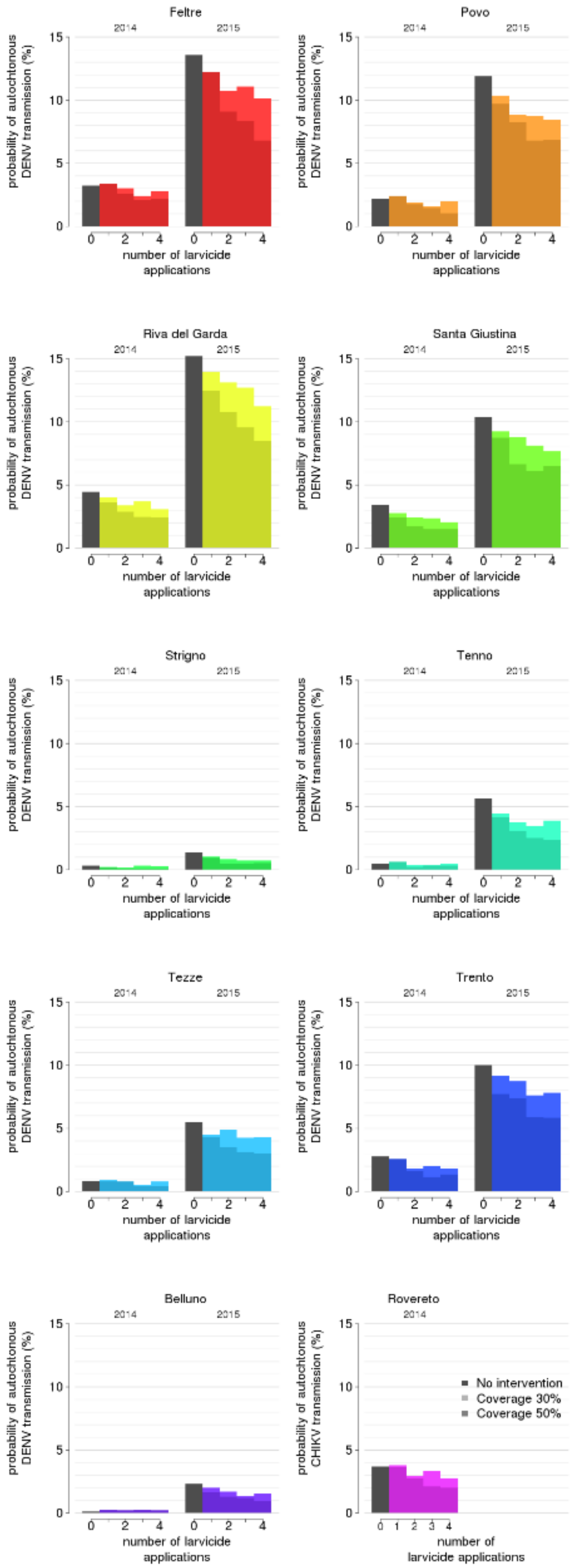

Figure S7.2. *Probability of local transmission with optimally timed interventions, for different coverages and effort levels, disaggregated by site and year.*

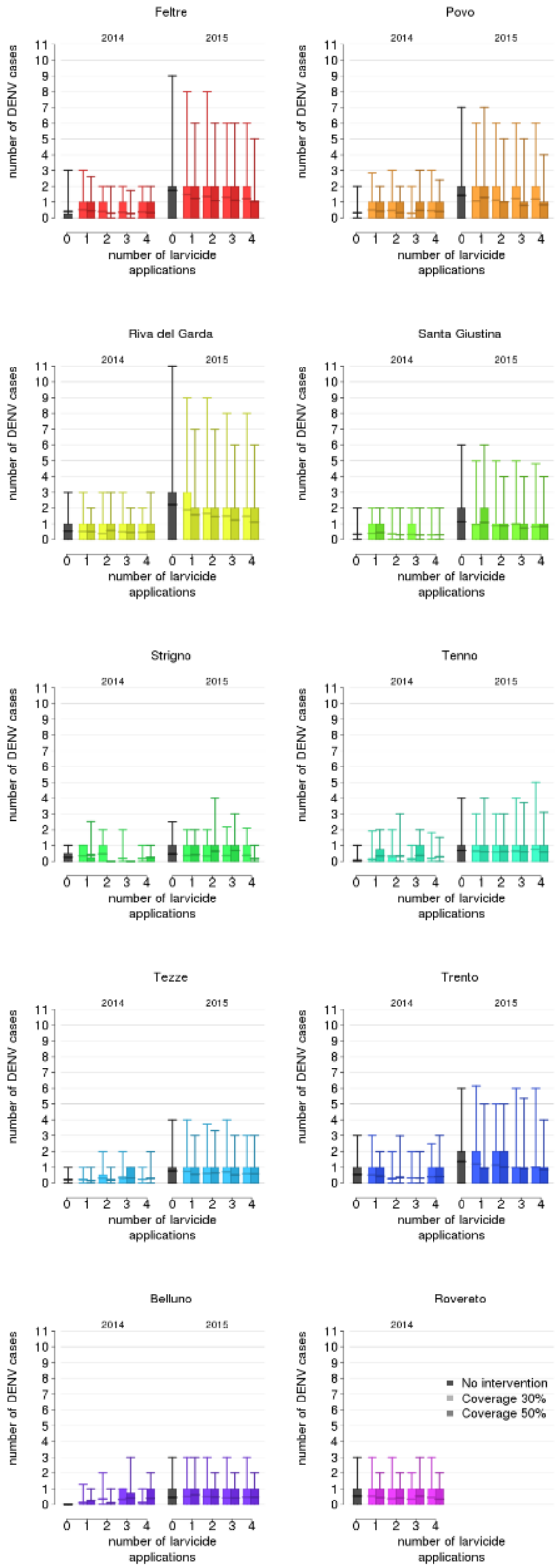

Figure S7.3. *Distribution of outbreak sizes with optimally timed interventions, for different coverages and effort levels, disaggregated by site and year.*

In the case of dengue, given that local transmission is limited to sporadic events, the reduction in the outbreak size afforded by larviciding is negligible, and the benefits of the intervention derive mainly from a reduction in the transmission probability.

8. Effect of larviciding in both public and private catch basins

In Figures S8.1-S8.3, we report results on the epidemiological effectiveness of larviciding in both public and private catch basins (as in Figures 2b and 3 in the main text for public-only interventions).

Figures S8.4-S8.6 shows the probability of optimal strategy in terms of net health benefit, comparing no intervention vs. public-only larviciding vs. public and private larviciding, assuming optimally timed scenarios with 2, 3 and 4 larvicide applications in a mosquito season (as in Figure 5 in the main text for single treatment).

9. Minimum average number of imported cases to observe secondary cases

Based on our estimates on the probability of outbreak *p* given an imported infection in a given site and year, we can define the probability *q* of not observing secondary cases after *n* arrivals of viraemic cases as: $q = (1-p)^n$. Therefore, for each site and year we can estimate the minimum number *N* of importations after which the likelihood of not observing a secondary case is below a given threshold *Q*:

$$N = \left] \frac{log(Q)}{log(1-p)} \right[$$

where the] . [operator represents the ceiling function (rounding to the first integer above). In table S9.1 we report the values of N for Q=10% and different infections, sites and treatment scenarios of public larviciding. Table S9.2 reports analogous values for larviciding in both public and private catch basins. To facilitate the interpretation of the tables, we colored the cells by risk levels defined by (arbitrary) thresholds on the number of required importations: the risk was considered high for N between 0 and 15, moderate between 16 and 30, low between 31 and 50 and negligible above or equal 51.

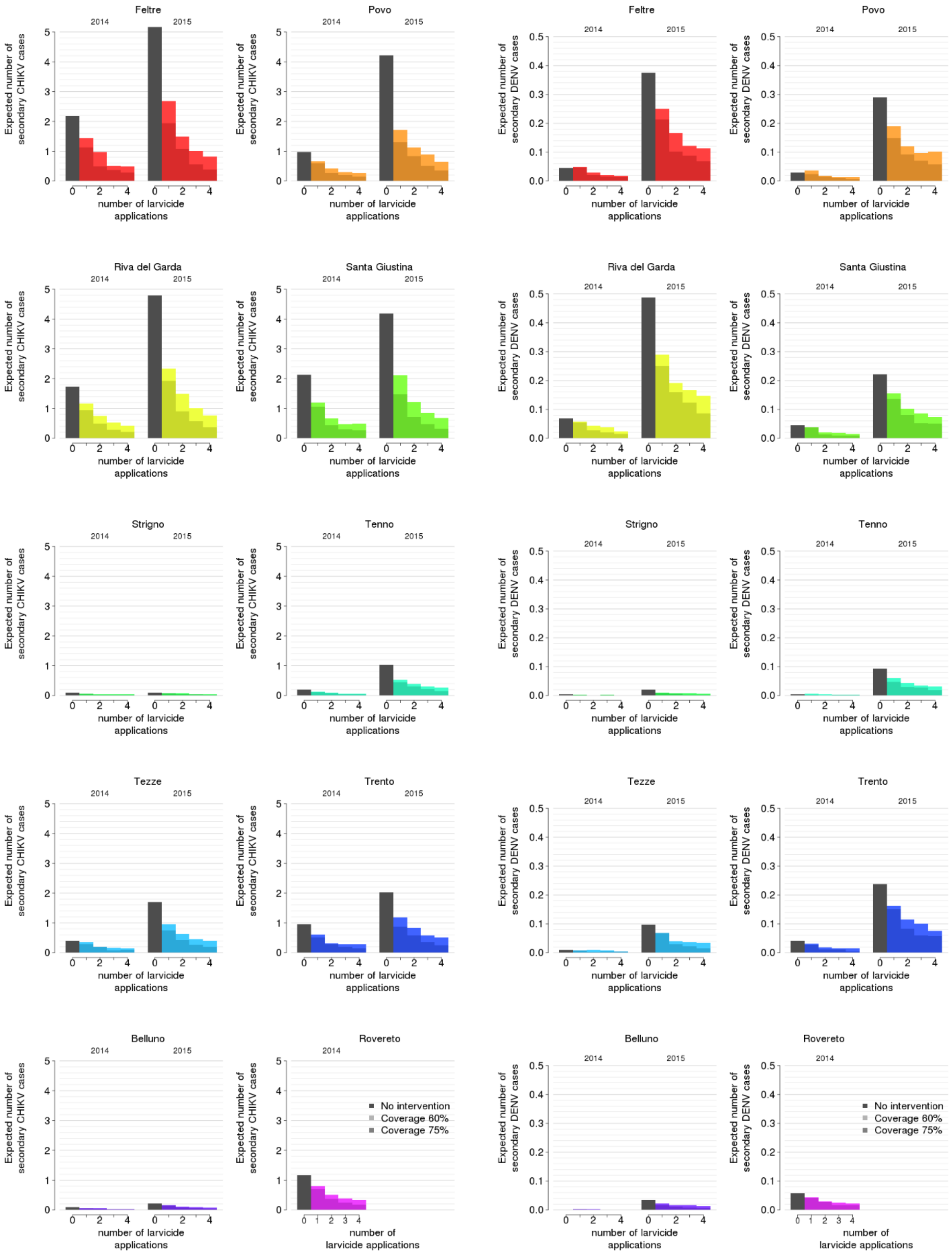

A) B)

Figure S8.1. *Expected number of A) chikungunya and B) dengue cases with optimally timed treatment of public and private catch basins, for different coverages and effort levels, disaggregated by site and year.*

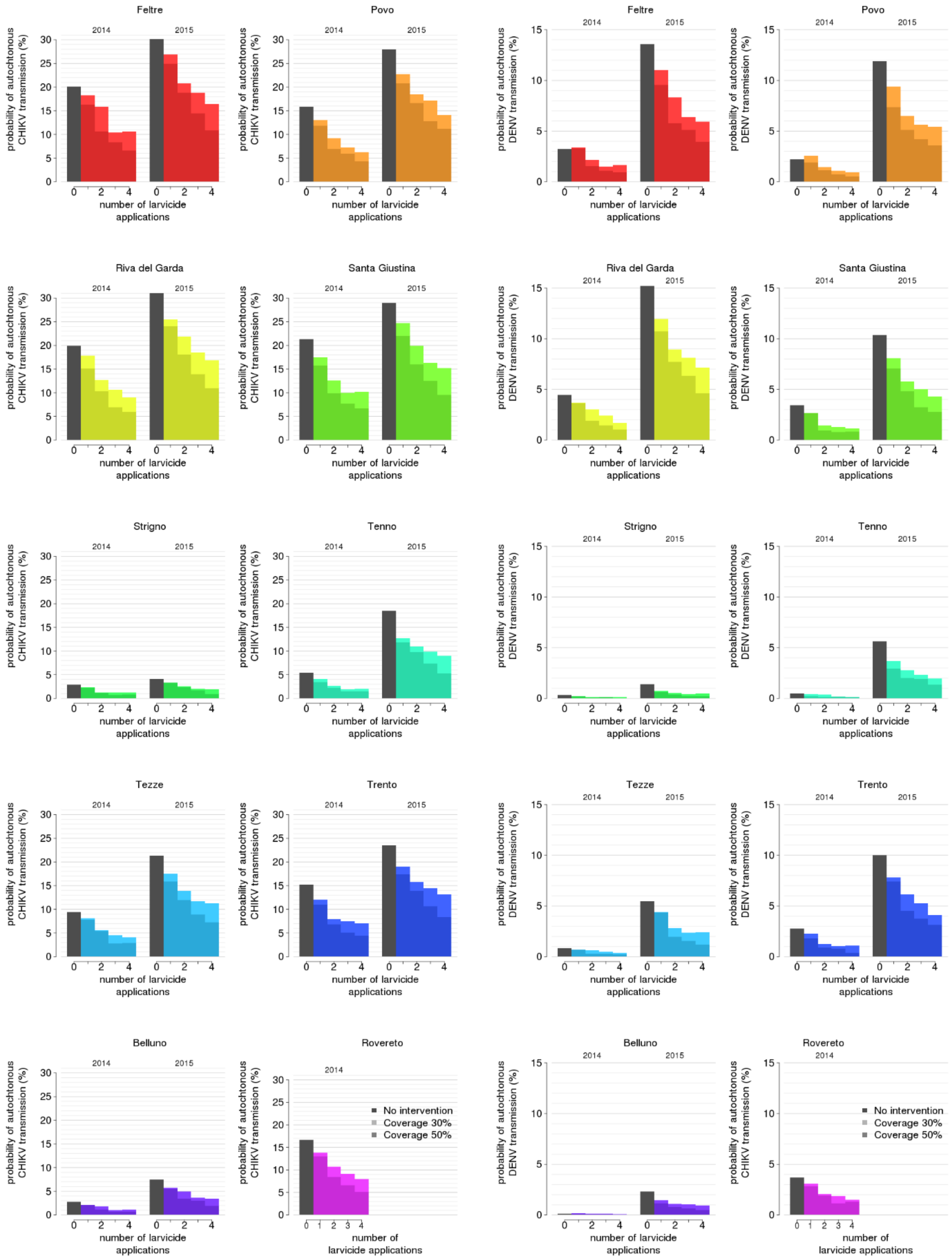

A)

B)

Figure S8.2. *Probability of local transmission for A) chikungunya and B) dengue with optimally timed treatment of public and private catch basins, for different coverages and effort levels, disaggregated by site and year.*

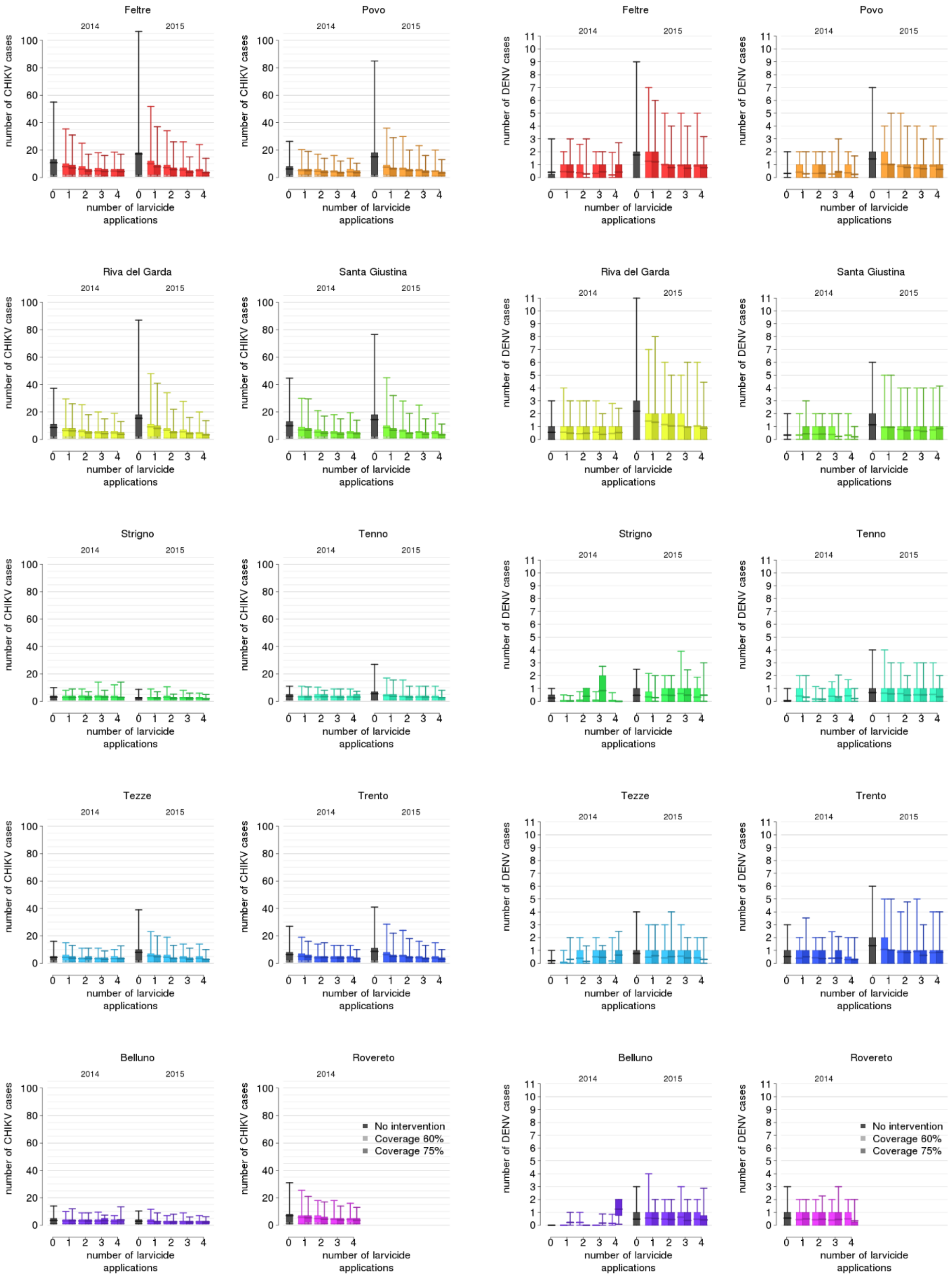

A) **B)**

Figure S8.3. *Distribution of outbreak sizes for A) chikungunya and B) dengue with optimally timed treatment of public and private catch basins, for different coverages and effort levels, disaggregated by site and year.*

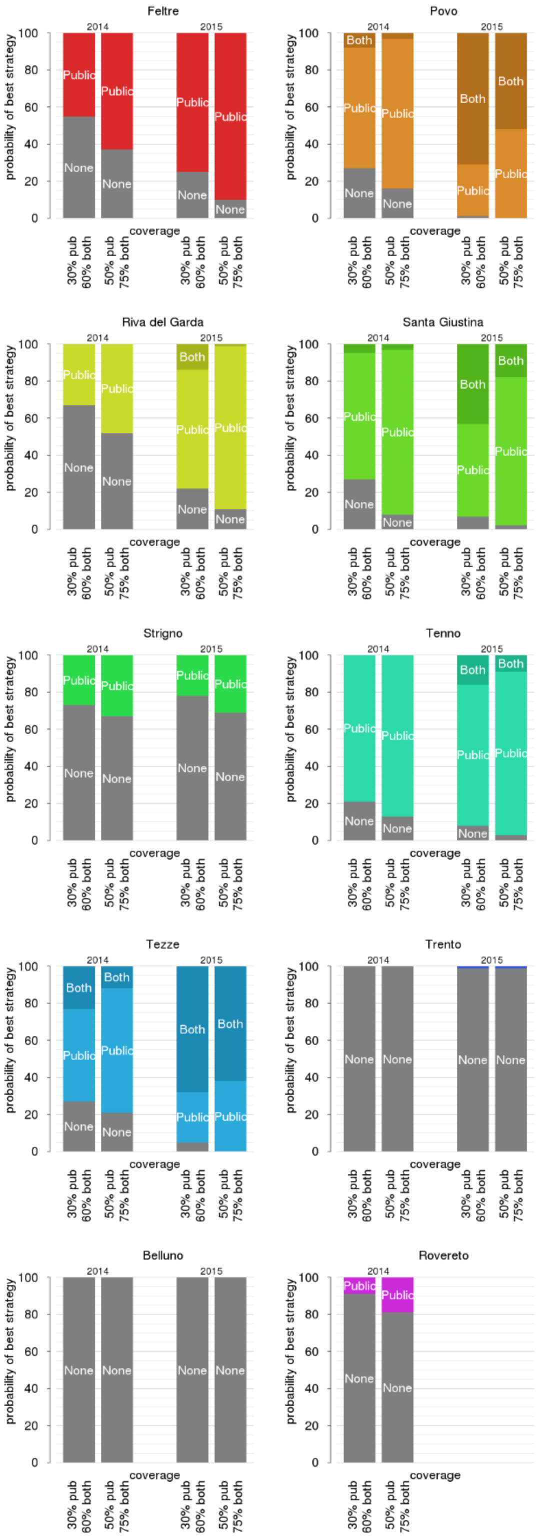

Figure S8.4. *Probability of producing the highest net health benefit by type of intervention (none vs. public vs. public and private) by year, coverage assumption and study site with 2 larvicide applications in a given season.*

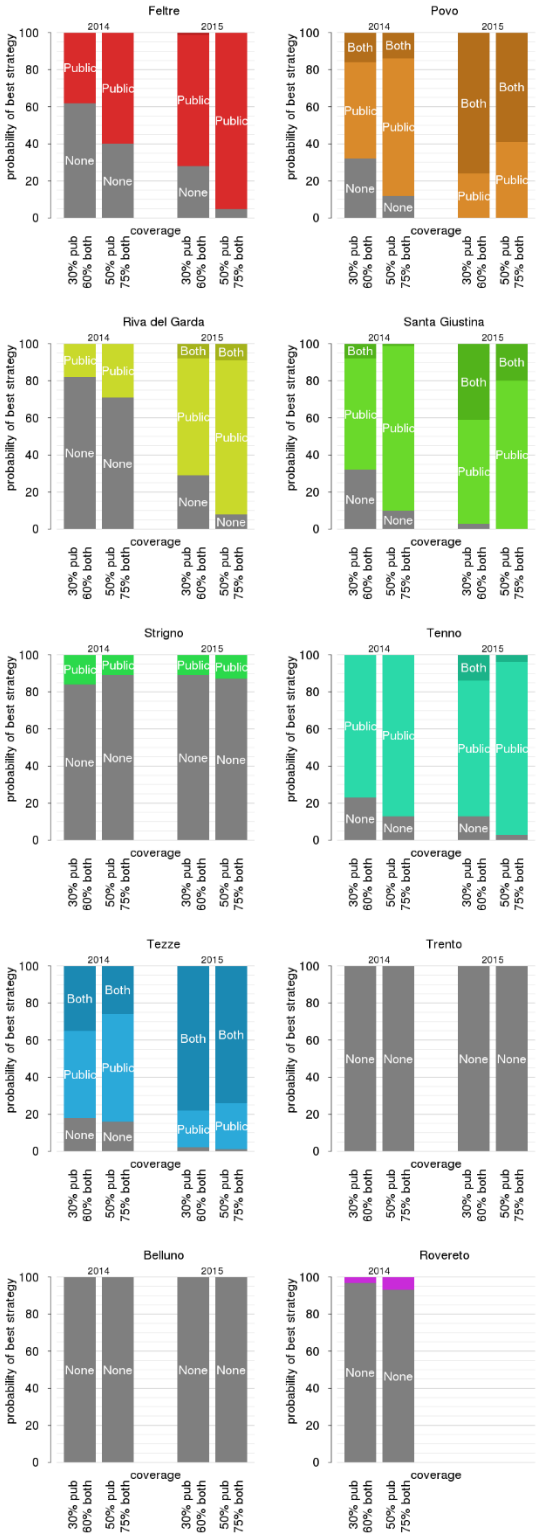

Figure S8.5. *Probability of producing the highest net health benefit by type of intervention (none vs. public vs. public and private) by year, coverage assumption and study site with 3 larvicide applications in a given season.*

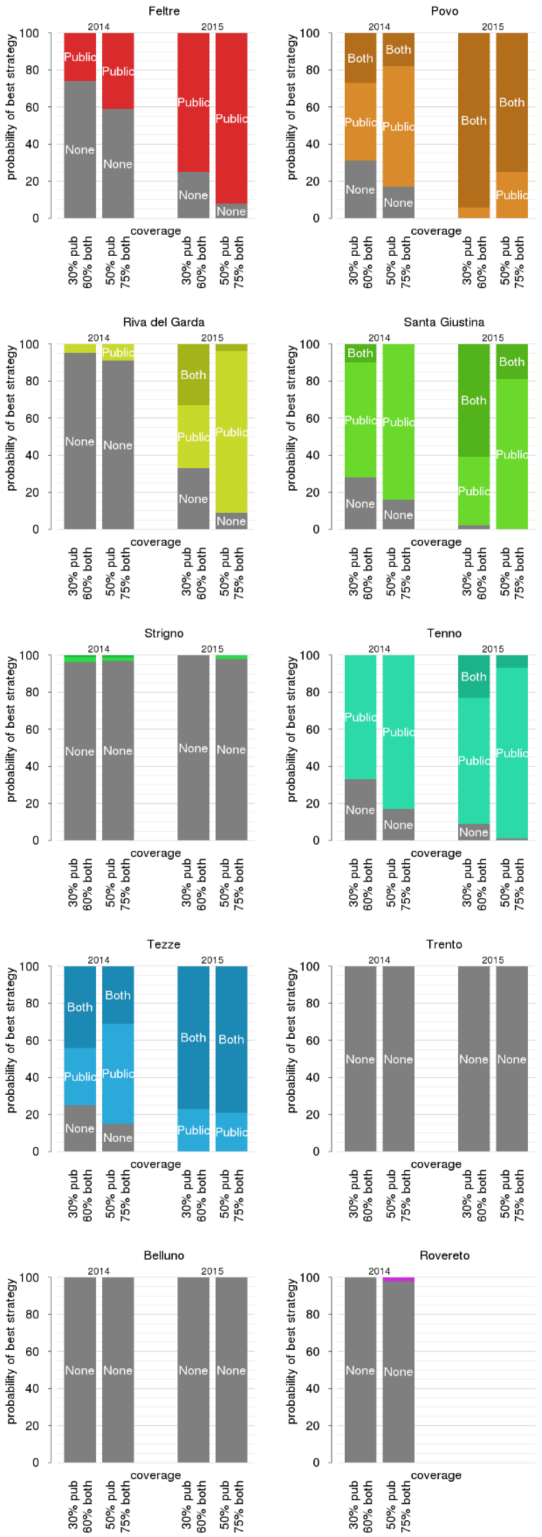

Figure S8.6. *Probability of producing the highest net health benefit by type of intervention (none vs. public vs. public and private) by year, coverage assumption and study site with 4 larvicide applications in a given season.*

Site	Chikungunya						Dengue					
	2014			2015			2014			2015		
	0%	30%	50%	0%	30%	50%	0%	30%	50%	0%	30%	50%
Feltre	11	12	18	7	8	11	70	85	111	16	20	29
Povo	14	18	25	8	10	12	104	119	181	19	25	31
Riva del Garda	11	15	19	7	9	11	51	67	90	14	20	25
Santa Giustina	10	14	19	7	9	12	66	97	158	22	29	37
Strigno	80	102	131	55	71	84	657	1030	1354	163	383	377
Tenno	42	60	88	12	17	22	469	556	758	40	60	90
Tezze	24	29	40	10	13	16	273	357	486	41	52	74
Trento	14	21	26	9	11	15	82	120	149	22	28	37
Belluno	82	111	145	30	40	55	1534	1579	1580	99	148	197
Rovereto	13	17	24	-	-	-	62	83	96	-	-	-

Table S9.1. Minimum number of imported cases for a >90% probability of secondary cases under no intervention and minimum (30%) and maximum (50%) coverages of larviciding in public catch basins repeated four times during the season. Orange: high risk (0-15 cases); yellow: moderate risk (16-30 cases); green: low risk (31-50 cases); blue: negligible risk (>50 cases).

.

Site	Chikungunya						Dengue					
	2014			2015			2014			2015		
	0%	60%	75%	0%	60%	75%	0%	60%	75%	0%	60%	75%
Feltre	11	20	31	7	13	18	70	138	227	16	34	51
Povo	14	21	49	8	15	21	104	196	341	19	40	60
Riva del Garda	11	23	34	7	13	19	51	120	172	14	30	41
Santa Giustina	10	22	31	7	14	20	66	192	230	22	45	68
Strigno	80	185	308	55	115	205	657	1569	5313	163	445	758
Tenno	42	112	149	12	25	40	469	1079	1328	40	101	153
Tezze	24	52	79	10	20	30	273	704	784	41	86	139
Trento	14	32	49	9	17	25	82	204	361	22	45	65
Belluno	82	210	309	30	66	109	1534	3289	8634	99	250	
Rovereto	13	28	40	-	-	-	62	146	172	-	-	-

Table S9.2. Minimum number of imported cases for a >90% probability of secondary cases under no intervention and minimum (60%) and maximum (75%) coverages of larviciding in both public and private catch basins repeated four times during the season. Orange: high risk (0-15 cases); yellow: moderate risk (16-30 cases); green: low risk (>30 cases); blue: negligible risk (>100 cases).

S1. Guzzetta G, Montarsi F, Baldacchino F, Metz M, Capelli G, Rizzoli A, Pugliese A, Rosà R, Poletti P, Merler S. Potential risk of dengue and chikungunya outbreaks in northern Italy based on a population model of Aedes albopictus (Diptera: Culicidae). PLos Negl Trop Dis. 2016.

S2. Poletti P, Messeri G, Ajelli M, Vallorani R, Rizzo C, Merler S. Transmission potential of chikungunya virus and control measures: the case of Italy. PLoS One. 2011;6(5):e18860.

S3. Carrieri M, Angelini P, Venturelli C, Maccagnani B, Bellini R. Aedes albopictus (Diptera: Culicidae) population size survey in the 2007 Chikungunya outbreak area in Italy. I. Characterization of breeding sites and evaluation of sampling methodologies. Journal of medical entomology. 2011;48(6):1214-25.

S4. Focks D, Daniels E, Haile DG, Keesling JE. A simulation model of the epidemiology of urban dengue fever: literature analysis, model development, preliminary validation, and samples of simulation results. The American journal of tropical medicine and hygiene. 1995; 53: 489–506.

S5. Lourenço J, Recker M. The 2012 Madeira dengue outbreak: epidemiological determinants and future epidemic potential. PLoS Negl Trop Dis. 2014; 8(8):e3083.

S6. Grange L, Simon-Loriere E, Sakuntabhai A, Gresh L, Paul R, Harris E. Epidemiological risk factors associated with high global frequency of inapparent dengue virus infections. Protective Immune Response to Dengue Virus Infection and Vaccines: perspectives from the field to the bench. 2015:14.

S7. Guilarde AO, Turchi MD, Siqueira Jr JB, Feres VC, Rocha B, Levi JE, Souza VA, Boas LS, Pannuti CS, Martelli CM. Dengue and dengue hemorrhagic fever among adults: clinical outcomes related to viremia, serotypes, and antibody response. Journal of Infectious Diseases. 2008;197(6):817-24.

S8. Global Burden of Disease 2004 update. Geneva.

S9. Suaya JA, Shepard DS, Siqueira JB, Martelli CT, Lum LC, Tan LH, Kongsin S, Jiamton S, Garrido F, Montoya R, Armien B. Cost of dengue cases in eight countries in the Americas and Asia: a prospective study. The American journal of tropical medicine and hygiene. 2009;80(5):846-55.

S10. Anderson KB, Chunsuttiwat S, Nisalak A, Mammen MP, Libraty DH, Rothman AL, Green S, Vaughn DW, Ennis FA, Endy TP. Burden of symptomatic dengue infection in children at primary school in Thailand: a prospective study. The Lancet. 2007;369(9571):1452-9.

S11. Luz PM, Grinsztejn B, Galvani AP. Disability adjusted life years lost to dengue in Brazil. Tropical Medicine & International Health. 2009;14(2):237-46.

S12. Moro ML, Gagliotti C, Silvi G, Angelini R, Sambri V, Rezza G, Massimiliani E, Mattivi A, Grilli E, Finarelli AC, Spataro N. Chikungunya virus in North-Eastern Italy: a seroprevalence survey. The American journal of tropical medicine and hygiene. 2010;82(3):508-11.

S13. LaBeaud A, Bashir F, King CH. Measuring the burden of arboviral diseases: the spectrum of morbidity and mortality from four prevalent infections. Population Health Metrics. 2011;9(1):1.

S14. Baldacchino F, Bussola F, Arnoldi D, Marcantonio M, Montarsi F, Capelli G, Rosà R, Rizzoli A. An integrated pest control strategy against the Asian tiger mosquito in northern Italy: a case study. Pest Management Science. 2017;73(1):87-93.
